# Anticancer mechanism of 7-α-hydroxyfrullanolide on microtubules and computational prediction of its target binding in triple-negative breast cancer cells

**DOI:** 10.7717/peerj.13508

**Published:** 2022-05-27

**Authors:** Siriphorn Chimplee, Carl Smythe, Varomyalin Tipmanee, Suchada Sukrong, Kanyanatt Kanokwiroon

**Affiliations:** 1 Department of Biomedical Sciences and Biomedical Engineering, Faculty of Medicine, Prince of Songkla University, Hat Yai, Songkhla, Thailand; 2 School of Biosciences, University of Sheffield, Sheffield, United Kingdom; 3 Center of Excellence in DNA Barcoding of Thai Medicinal Plants, Department of Pharmacognosy and Pharmaceutical Botany, Faculty of Pharmaceutical Sciences, Chulalongkorn University, Bangkok, Thailand

**Keywords:** 7-α-Hydroxyfrullanolide, Sesquiterpene lactones, Natural product, Triple-negative breast cancer, Microtubules, G2/M arrest, G1 arrest, DNA damage response, Molecular docking

## Abstract

**Background:**

Triple-negative breast cancer (TNBC) responds poorly to the available drugs; thus, the mortality rate associated with TNBC remains high. 7-α-Hydroxyfrullanolide (7HF) possesses anticancer properties and arrests cells in the G2/M-phase via modulation of several proteins involved in the G2/M-phase transition, as well as the mitotic checkpoint in MDA-MB-468 (TNBC) cells. Microtubules (MTs) dynamically regulate cell division in the G2/M phase and are related to cancer cell stress response. However, antimitotic drug cytotoxicity to multiple cancer resistance developed in response to drugs are obstacles faced to date. Here, the activity and mechanism via which 7HF controls MTs dynamics was investigated in MDA-MB-468 cells.

**Methods:**

7HF uptake by MDA-MB-468 cells was assessed using spectrophotometry. The drug-like properties of 7HF were predicted using the Swiss-absorption, distribution, metabolism, and excretion (ADME) webtool. Then, the effect of 7HF treatment (6, 12, and 24 µM) on the dynamic arrangement of MTs was assessed for 1, 12, and 24 h using indirect immunofluorescence. Polymerization of α- and β-tubulin was assessed using different 7HF concentrations in a cell-free system for 1 h. Cell proliferation assay with bromodeoxyuridine plus propidium iodide staining and flow cytometry was performed at different 7HF concentrations and time points. The mechanism of action was assessed by detecting the expression of proteins, including Bub3, cyclin B1, p-Cdk1 (Tyr15), Rb, p-Rb (Ser780), Chk1, p-Chk1 (Ser345), Chk2, p-Chk2 (Ser516), and p-H2AX (Ser139), using western blotting. Molecular docking was used to predict the molecular interactions between 7HF and tubulins in MTs.

**Results:**

We observed that 7HF was able to enter the MDA-MB-468 cells. The ADME webtool analysis predicted that it possesses the high passive permeation and gastrointestinal absorption properties of drugs. Various concentrations of 7HF disrupted the dynamic arrangement of spindle MTs by causing radial spindle array shrinkage and expansion of fibrous spindle density and radial array lengths in a time-dependent manner. 7HF reduced polymerization of α-, β-tubulin in dose-dependent manner. 7HF also triggered DNA damage response by inducing G2/M and G1 phase arrests in a concentration and time-dependent manner, which occurred due to the upregulation of Bub3, Chk1, p-Chk1 (Ser345), p-Cdk1 (Tyr15), and cyclin B1. According to molecular docking analysis, 7HF preferred to bind to β-tubulin over α-tubulin. The lactone, ketone, and hydroxyl groups of 7HF supported the 7HF-tubulin interactions. Hydrogen bonding with a hydrocarbon ring and salt bridge attractive forces were responsible for the binding versatility of 7HF.

**Conclusions:**

This is the first study to investigate the molecular mechanism, MTs interacting sites, and the internalization and drug-like properties of 7HF in TNBC cells. The findings will be useful for developing 7HF-based treatment for patients with TNBC.

## Introduction

Female breast cancer is the most common (2.3 million new cases) and the fifth leading cause of cancer-related mortality (0.68 million cases) worldwide, according to GLOBOCAN 2020 (Ferlay et al., 2021; Sung et al., 2021). Triple-negative breast cancer (TNBC) accounts for 15–20% of all breast cancer cases (Nunez Abad et al., 2021). TNBC exhibits poor biological behavior, with more aggressiveness, earlier recurrence, and ability to metastasize to distant organs than non-TNBC subtypes because of the lack of available therapeutic targets, such as estrogen receptor, progesterone receptor, and human epidermal growth factor receptor 2 (Nunez Abad et al., 2021). Therefore, the development of targeted therapies for patients with TNBC has been challenging (Nunez Abad et al., 2021). The high response rate to standard chemotherapy combinations (anthracycline taxanes with vinca alkaloid or targeted drugs) appears to improve the pathological complete response of a neoadjuvant or adjuvant regimen; however, an early recurrence of TNBC is high in such cases due to poor efficacy of prolonged chemotherapy (Bianchini et al., 2021; Nunez Abad et al., 2021; Wang et al., 2018). Thus, chemotherapy in TNBC is associated with low survival, high rate of relapse, high risk of secondary cancers, and strong adverse effects (De Ruijter et al., 2011; Liao, Apaya & Shyur, 2013; Nunez Abad et al., 2021; Sharma, 2016; Sharma, 2018).

7-α-Hydroxyfrullanolide (7HF), a natural product, is a eudesmanolide sesquiterpene lactone (SL) and is isolated from the flowering plants of the Asteraceae family, such as *Grangea maderaspatana* L. Poir. and *Sphaeranthus indicu*s Linn. (Nahata et al., 2013; Pandey et al., 2019; Ruangrungsi et al., 1989). 7HF is used to produce its bioactive derivatives *via* fungal transformation (Atta ur et al., 1994). It also inhibits DNA topoisomerase I (Top I) in yeast cells and possesses anti-inflammatory and anticancer activities (Fonseca et al., 2010; Nahata et al., 2013; Pandey et al., 2019; Uppatanpreecha, 2009). It inhibits the proliferation of certain types of cancer cells, such as small cell lung cancer, oral cavity cancer, colon cancer, and breast cancer cells (Fonseca et al., 2010; Nahata et al., 2013; Pandey et al., 2019; Uppatanpreecha, 2009). A previous study on the anti-breast cancer activity of 7HF showed that TNBC cells (MDA-MB-468, MDA-MB-231, and Hs578T) are more sensitive to 7HF than non-TNBC cells (MCF-7) (Chimplee et al., 2022). 7HF has been reported to be associated with G2/M arrest and apoptosis signaling pathways in TNBC (MDA-MB-468) and colon cancer cells (Chimplee et al., 2022; Nahata et al., 2013; Pandey et al., 2019). Notably, 7HF triggers G2/M arrest by modulating the expression of several proteins involved in the regulation of G2/M phase transition, mitotic chromosome segregation, mitotic checkpoint regulation, and mitotic spindle organization in MDA-MB-468 cells (Chimplee et al., 2022). Mitotic associated proteins of tubulins and Bub3 mitotic checkpoint were significantly upregulated by 7HF in the TNBC cells indicating through controlling of MTs formation (Chimplee et al., 2022). Hence, the additional molecular mechanism of the anticancer activity of 7HF on MTs in TNBC cells should also be elucidated.

The microtubule (MT) cytoskeleton is a highly dynamic structure (Avila, 1992). MTs consist of α-, β-tubulin subunits and play a critical role in cell division, cell movement, and intracellular signaling (Mollinedo & Gajate, 2003). MT stress induces G2/M phase cell cycle arrest, which is one of the mechanisms of action of antimitotic drugs used in cancer treatment, such as taxanes and vinca alkaloids (Gascoigne & Taylor, 2009; Parker, Kavallaris & McCarroll, 2014; Tischer & Gergely, 2019). In addition, sesquiterpene lactones (SLs) exhibit anti-MT activity by regulating MT dynamics, and the proposed mechanism of action involves a target protein associated with an arresting of G2/M or M-phases in many cancer cells (Bosco & Golsteyn, 2017). SLs, including parthenolide, costunolide, artesunate, santamarine, calein C, 6-O-angeloylplenolin, coronopilin, pulchelloid A, and hymenoratin, have shown promising anti-MT activity by inducing G2/M or M phase arrest in tested cancer cell lines (Bosco & Golsteyn, 2017; Bosco et al., 2021; Caldas et al., 2018; Cotugno et al., 2012; Dumontet & Jordan, 2010; Liu et al., 2011a; Liu et al., 2011b; Manchado, Guillamot & Malumbres, 2012; Molina et al., 2021; Steinbruck, Pereira & Efferth, 2010; Whipple et al., 2013). The anti-MT activity of SLs has been studied extensively and their mechanism of action has been reported; however, the specific overcome to target MT-binding is not sufficient (Bosco & Golsteyn, 2017; Gallagher & Brian, 2007).Therefore, studies for the development of new MT-binding agents that can be used for the development of TNBC-targeted drugs are in progress. Internalization and accumulation of drugs in cancer cells are also crucial for improving the chemotherapeutic efficacy (Nunez Abad et al., 2021; Piccolo et al., 2019). Hence, the stability of drugs in TNBC cells should also be assessed.

In this study, we aimed to investigate the effects of 7HF on MT dynamics and its molecular mechanism of action. Cellular uptake of 7HF, MT-7HF interaction, and the drug-like properties of 7HF were also determined.

## Materials and Methods

### Plant material preparation and 7HF isolation

**Figure 1:**
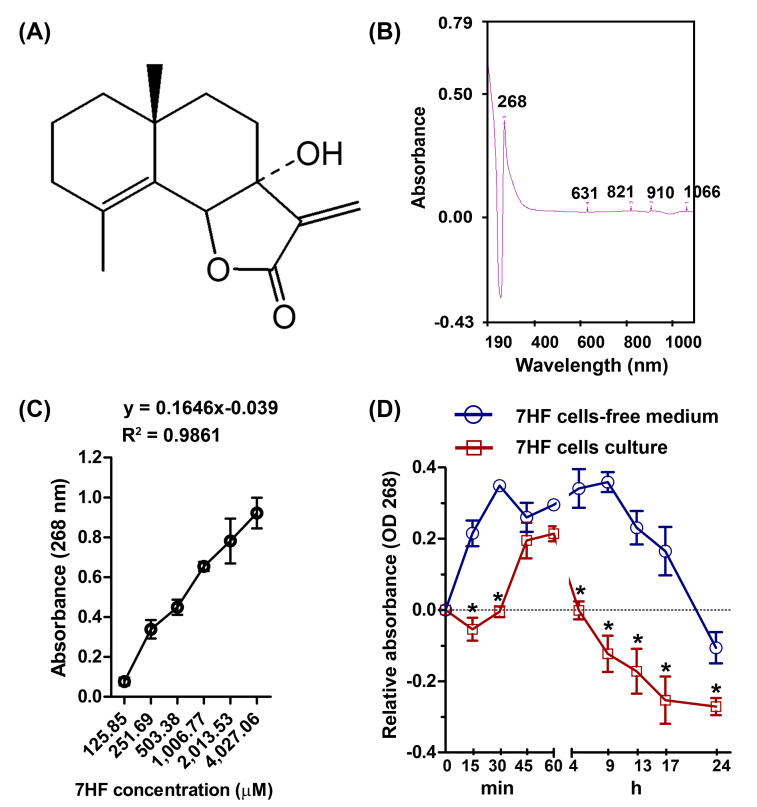
7-α-Hydroxyfrullanolide (7HF) uptake by MDA-MB-468 cells. (A) Chemical structure of 7HF. (B) The identical peaks of 7HF at 268 nm and 1,006.77 µM. (C) 7HF calibration curve at 268 nm. 7HF concentration ranged from 125.85 to 4,027.06 µM. (D) 7HF uptake profile of MDA-MB-468 cells compared to that of the control (7HF-cells-free medium). Mean differences were analyzed using Two-way ANOVA and Bonferroni posttests multiple comparisons between cells- and cells-free cultures (*p < 0.001). The amount of 7HF was subtracted from that present in only medium and DMSO-medium (in cells-free medium) and in cells-medium and DMSO-cells medium (in cells culture).

Dried *G. maderaspatana* (L.) Poir plants were purchased from the Chaokromper Drug Store (Bangkok, Thailand). The plant material was prepared, and 7HF was isolated as described previously (Chimplee et al., 2022). The chemical structure of 7HF is shown in Fig. 1A. 7HF was stored at −20 °C.

### Cancer cell culture

The MDA-MB-468 cells were purchased from the American Type Culture Collection (ATCC, Manassas, VA, USA) and cultured in Dulbecco’s modified Eagle’s medium (DMEM) (Gibco; Thermo Fisher Scientific, Inc., Waltham, MA, USA) with condition as described previously (Chimplee et al., 2019; Chimplee et al., 2022).

### 7HF uptake assay

7HF (125.85–4,027.06 µM) dilutions in dimethyl sulfoxide (DMSO) were scanned to identify the maximum absorbance (*λ*_max_) between 190 and 1,100 nm, using a UV-visible spectrophotometer (Shimadzu, Kyoto, Japan). The *λ*_max_ was obtained at 268 nm and at a concentration of 1,006.77 µM (Fig. 1B and Fig. S1). To test whether 7HF was able to pass through the TNBC cells, the cells were treated with 1,006.77 µM 7HF. MDA-MB-468 cells (3 × 10^4^ cells/well) were seeded on 96-well plate and incubated with the DMEM medium overnight. Then, 100 µL of 1,006.77 µM 7HF was used to treat the TNBC cells for 0.25–24 h at 37 °C in a 5% CO_2_ incubator. In case of 7HF cells-free medium, 1,006.77 µM of 7HF was prepared with only medium. After incubation, the remaining of 7HF supernatant in cells culture medium and cells-free medium was immediately collected without washing with buffer. The absorbance of 7HF was then measured at 268 nm and the absorbance was plotted *versus* time (Fig. 1D and Table S2). The mean absorbance values of 7HF were subtracted from the absorbance value of only medium and DMSO-supplemented medium (in cells-free medium), and cells medium and cells-DMSO medium (in cells-culture). The experiment was performed in triplicate. In addition, a concentration curve of 7HF was plotted on a linear scale at 268 nm and concentrations ranging from 125.85 to 4,027.06 µM (Fig. 1C and Table S1). The linear regression coefficients were calculated using the general equation of the calibration curve (*y* = *ax* + *b*; *a* = 0.1646, *b* =  − 0.039, and *R*^2^ = 0.9861) (Fig. 1C).

### Immunofluorescence (IF) assay

The effect of 7HF on MT dynamics in the TNBC cells was visualized using indirect IF, with certain modifications (Duangmano et al., 2012). In brief, MDA-MB-468 cells (1.5 × 10^5^ cells/well) were seeded on cover slips and incubated at 37 °C in a 5% CO_2_ incubator overnight. After washing with phosphate-buffered saline (PBS), the TNBC cells were treated with 6, 12, and 24 µM 7HF for 1, 12, and 24 h. The 7HF concentrations were obtained from 0.5X, 1X, and 2X its IC_50_ by MTT assay at 72 h, as reported previously (Chimplee et al., 2022). Paclitaxel (PTX), vincristine (VCR), and doxorubicin (DOX), 200 nM each, were also used to treat the TNBC cells for 24 h, with minor adaptation (Blajeski et al., 2002; Mukhtar, Adhami & Mukhtar, 2014). The control was untreated cells with indicated concentrations of 7HF and chemotherapies. Then, the cells were fixed with 4% formaldehyde, permeabilized with 0.5% Triton X-100, and blocked in 1% bovine serum albumin (BSA) in PBS. After washing the cover slips, the untreated and treated cells were stained overnight with anti- α-tubulin antibody (Cell Signaling Technology, Inc., Danvers, MA, USA; 1:50) and DAPI (Sigma-Aldrich, St. Louis, MO, USA; 1:1,000), followed by incubation with Alexa Fluor 488-conjugated goat anti-IgG rabbit secondary polyclonal antibody (GE Healthcare Ltd., Little Chalfont, Bu, UK; 1:200) for 1 h. Finally, the stained coverslips were mounted with 50% glycerol and sealed on a glass slide. The slides were visualized under an inverted fluorescence microscope (EVOS, Thermo Fisher Scientific Inc.).

### Tubulin polymerization assay in cell-free system

The effects of 7HF on tubulin polymerization were analyzed using the tubulin polymerization assay kit (Cytoskeleton Inc., Denver, CO, USA). The assay was performed following the manufacturer’s instructions. Briefly, 2 mg/mL tubulin was diluted using tubulin polymerization buffer. After pre-warming of empty dark 96-well plate at 37 °C in a Spark microplate reader (Tecan, Männedorf, Switzerland) for 5 min, 5 µL 7HF (6, 12, and 24 µM), PTX (3 µM), VCR (3 µM), DOX (3 µM), and tubulin control were added, and the plate was incubated for 1 min, after which 50 µL of tubulin reaction reagent was manually injected into the microplate reader immediately. The effect of reagents on tubulin polymerization was then measured at 360 and 450 nm at 37 °C, every minute up to 1 h in kinetic mode.

### Cell proliferation assay using fluorescence-activated cell sorting (FACS)

To study the effect of MT dynamics on DNA damage in 7HF-treated cells, we first treated TNBC cells with anti-bromodeoxyuridine (BrdU) and then incubated them with propidium iodide (PI) to stain the newly synthesized DNA. The protocol for this experiment was adapted from Cecchini, Amiri & Dick (2012) and Zhu (2012), with some modifications. The MDA-MB-468 cells (1 × 10^6^ cells/plate) were cultured and pulsed with 25 µM BrdU (Cayman Chemical, Ann Arbor, MI, USA) for 30 min. Untreated and 7HF-treated cells (6, 12, and 24 µM) were harvested after 12 and 24 h. Then, 1 × 10^6^ cells were counted. After centrifugation (7,000 rpm, 5 mins), the cell pellets were resuspended in 2 mM EDTA, fixed with 70% EtOH, and stored overnight at −20 °C. Then, the cells were harvested and resuspended in washing buffer (0.5% BSA in PBS). After centrifugation (10,000, 2 mins), the cell pellets were resuspended in 2 M HCl and sodium borate (pH 8.5) solution and incubated for 30 min. The cell pellets were washed with washing buffer containing 0.2% Tween-20. After centrifugation (10,000 rpm, 2 mins), anti-BrdU primary antibody (20 µL) and RNase (200 µg/mL) were added, and the cells were incubated overnight at 4 °C. Then, Alexa Fluor 488-conjugated goat-anti-IgG mouse secondary antibody (Thermo Fisher Scientific, Inc.; 50 µL of 50 µg/mL) and PI (Sigma-Aldrich; 20 µg/mL) were added, and the cells were incubated for 1 h prior to injection in the FACS machine (Becton Dickinson, San Jose, CA, USA). Density plots were generated using the CellQuestPro software (Becton Dickinson).

### Western blot analysis

To determine the effect of 7HF on the expression of proteins involved in MT dynamics and DNA damage response (DDR) pathways, MDA-MB-468 cells (3 × 10^5^ cells/well or 1 × 10^6^ cells/plate) were grown at 37 °C in a 5% CO_2_ incubator overnight. The cells were treated with 6 µM 7HF for 0, 12, 24, and 48 h. For optimizing 7HF concentration, the cells were incubated for 24 h in the presence of 0, 6, (or 12), and 24 µM 7HF. Chemiluminescence-based western blotting was performed as described previously (Chimplee et al., 2022). Briefly, 50 µg protein was separated electrophoretically 12% sodium dodecyl sulfate (SDS)-polyacrylamide gel electrophoresis (PAGE) and transferred onto nitrocellulose membrane (Chimplee et al., 2022). The blots were blocked and incubated overnight with 1;1,000 dilutions of various primary antibodies (anti-cyclin B1, anti-p-Cdk1 (Tyr15), anti-Bub3, and anti-β-actin; Cell Signaling Technology, Inc.) at 4 °C. After washing, the blots were incubated with horseradish peroxidase-conjugated anti-rabbit IgG (GE Healthcare Ltd; 1:5,000) for 1 h.

For fluorescence-based western blotting, the protein samples were extracted using radioimmunoprecipitation assay buffer (Tris pH 7.5, NaCl, EDTA, EGTA, NP-40, sodium deoxycholate, sodium pyrophosphate, β-glycerophosphate, and protease inhibitor cocktail) and quantified using the Bradford assay (Bio-Rad Laboratories, Hercules, CA, USA). Then, 50 µg protein was separated on SDS-PAGE gels (8%, 12%, and 15% variation with molecular weight of protein) and blotted onto nitrocellulose membranes at 100 V for 2 h. Apart from phosphoproteins for which BSA was used for blocking, the blots were blocked with blocking buffer (5% low-fat dry milk in Tris buffered saline) for 1 h. After washing, the blots were incubated with various primary antibodies for Rb, p-Rb (Ser780), Chk2, p-Chk2 (Ser516), and β-actin (Cell Signaling Technology, Inc.), Chk1, p-Chk1 (Ser345) (kindly provided by Prof. Dr. Carl Smythe), and p-H2AX (Ser139) (EMD Millipore Corp., Billerica, MA, USA) overnight 4 °C. The following antibody concentrations were used: 1:500 for anti-p-Chk2, 1:1,000 for anti-p-Rb, anti-Chk1, anti-p-Chk1, and anti-p-H2AX, 1:2,500 for anti-Rb and anti-Chk2, and 1:5,000 for anti-β actin. The blots were incubated for 1 h in 1:1,000 dilutions of secondary IgGs with various hosts and fluorophore labels, including anti-sheep (for 680 nm), anti-mouse (for 800 nm), and anti-rabbit (for 680 nm) antibodies (LI-COR Biosciences, Lincoln, NE, USA). Finally, the protein bands were observed using a chemiluminescent detection kit (Thermo Fisher Scientific, Inc.) and the Fusion FX western blot imaging system (Vilber Lourmat Sté, Collégien, France). The relative protein expression was quantified using the Fusion Capt Advanced Quantitation Analysis program (Vilber Lourmat Sté). Fluorescence-based protein bands were observed using the Odyssey imaging system (LI-COR Biosciences) and relative expression was calculated using the Image Studio Lite 5.2 program (LI-COR Biosciences). Two-three independent experiments were performed.

### Prediction of 7HF drug-likeness

To predict the 7HF drug design, 7HF structure (PubChem CID 11983230) was submitted to the Swiss-absorption, distribution, metabolism and excretion-(ADME) web tool (http://www.swissadme.ch, accessed on 07 December 2021) (Daina, Michielin & Zoete, 2017).

### Molecular docking analysis

Molecular docking was done to predict whether 7HF bound to the target protein, as per a previous study with certain modifications (Saechan et al., 2021). The PDB ascension number 5SYF represents the three-dimensional (3D) protein structure of α-, β-tubulin (Kellogg et al., 2017). The AutoDock Tool (ADT) 4.1 was used to eliminate all water molecules. The other co-crystallized ligands were retained. The PDB file format contains all polar hydrogen atoms that emulate hydrogen bond interactions. The 3D structures of 7HF (CID 11983230), pironetin (CID 6438891), PTX (CID 36314), and VCR (ID 5758) were obtained from the PubChem and ChemSpider databases. The SMILES Translator and Structure File Generator (https://cactus.nci.nih.gov/translate/) created PDB files for 7HF, pironetin, PTX, and VCR. All polar hydrogen atoms were added using ADT. Finally, the protein and ligand structures were saved as PDB and Partial Charge (Q) and Atom Type (T) or PDBQT files.

All ligands were docked with the tubulin proteins using AutoDock4 version 4.2 (Morris et al., 2009). Using the SG atom of 315^th^-cysteine residue (Cys315) as the interaction point with pironetin, the grid box was centered at an *x*-*y*-*z* position of “329.387 449.699 378.122” with a grid size of 90 × 90 × 90 cubic angstrom (Å^3^) (Prota et al., 2016; Steinmetz & Prota, 2018; Yang et al., 2016). The grid box center of PTX and VCR was as per previous reports (Field Jessica, Díaz José & Miller John, 2013; Gigant et al., 2005). In total, 50 docking runs with a population of 200 were required. The default parameters of the AutoDock4 program were used. The quintuple independent samplings reported the lowest binding energy (Δ*G*_bind_). The drug-tubulin complex structure was visualized using the Visual Molecular Dynamics (VMD) package (Humphrey, Dalke & Schulten, 1996). The ligand-protein interactions were then studied and visualized using the Protein-Ligand Interaction Profiler (PLIP) webtool (Adasme et al., 2021).

### Statistical analysis

Each experiment was performed thrice. Data were analyzed using one-way analysis of variance (ANOVA) and two-way ANOVA using GraphPad Prism 5 (GraphPad Software, San Diego, CA, USA). The differences were considered significant at *p* < 0.05.

## Results

### 7HF uptake in MDA-MB-468 cells

In cells-free medium, 7HF was stable from 15 min to 17 h, and the stability gradually reduced up to 24 h (Fig. 1D and Table S2). In contrast, 7HF could not be detected between 5 h and 17 h in the cells culture medium (Fig. 1D and Table S2). 7HF may have penetrated MDA-MB-468 cells within 5 h and was internalized by 12 h.

### Effect of 7HF on microtubule dynamics, tubulin polymerization, and protein expression

**Figure 2:**
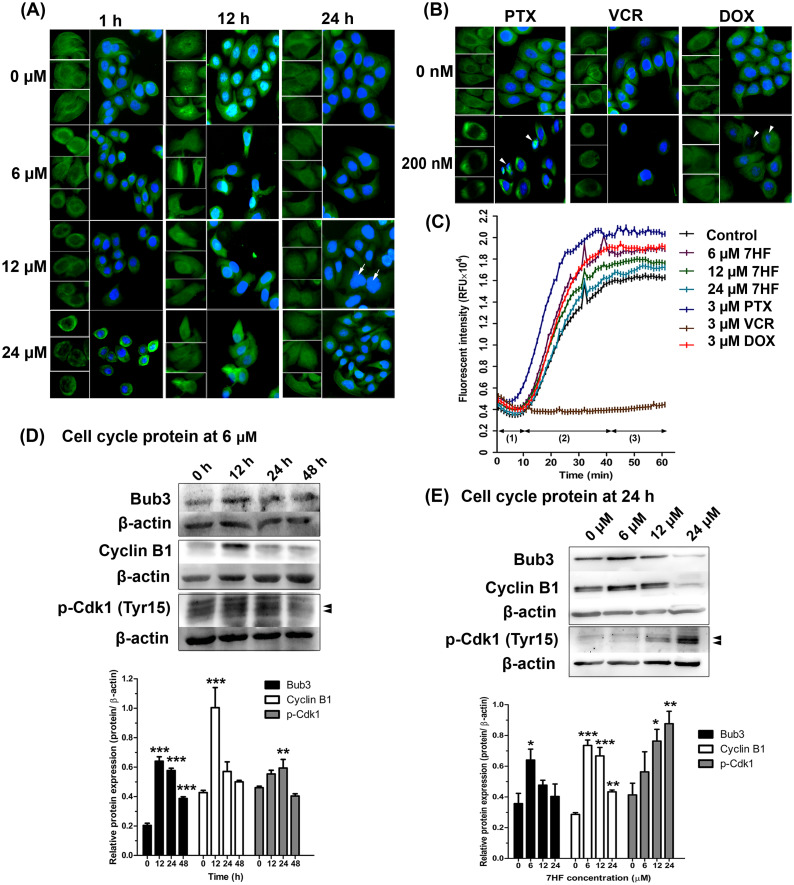
Effect of 7-α-hydroxyfrullanolide (7HF) on microtubule dynamics and protein expression in MDA-MB-468 cells. (A) Immunofluorescence (IF) of cells treated with 0, 6, 12, and 24 µM 7HF for 1, 12, and 24 h. (B) IF of cells treated with 0 and 200 nM PTX, VCR, and DOX for 24 h. PTX, paclitaxel; VCR, vincristine; DOX, doxorubicin; green, α-tubulin; blue, nuclear DNA; white arrow, likely to be M-phase cells; white head arrow, apoptotic cells; scale, 100 µm. (C) Results of tubulin polymerization assay performed using different 7HF concentrations (6 µM; purple, 12 µM; green, and 24 µM; light blue), and 3 µM chemotherapeutics (PTX, dark blue; VCR, brown, and DOX, red) for 1 h in cell-free system. (1) nucleation, (2) growth, and (3) steady state equilibrium during tubulin formation. RFU: Relative fluorescence units. (D) Bub3, cyclin B1, and p-Cdk1 (Tyr15) levels in cells treated with 6 µM 7HF for 0, 12, 24, and 48 h. (E) Bub3, cyclin B1, and p-Cdk1 (Tyr15) levels in cells treated with 0, 6, 12, and 24 µM 7HF for 24 h. Mean differences were statistically analyzed using one-way ANOVA and Bonferroni’s multiple comparison test. *p < 0.05, **p < 0.01, ***p < 0.001 compared to control (0 h or 0 µM). Black arrowhead, an indicated protein band.

To determine the effect of 7HF on spindle MT arrangement, α-tubulin staining (shown in green) was visualized using IF (Fig. 2A). We observed that the spindle MT arrangement differed from that of the control when treated with 6–24 µM 7HF, excepting at 24 h. The radial array of the spindle gradually reduced after 1 h. The fibrous density along the spindle length was differed between control and 12 h 7HF. Radial lengthening of the spindle array was insignificantly observed from that of control at 24 h. We also observed time-dependent changes (1–24 h), *i.e.,* the spindle MT length ranged from short radial arrays to long fiber when treated with 6–24 µM 7HF. Notably, maximum reduction in the number of α-tubulin-presenting cells was observed at 12 h for all 7HF concentrations. Spindle length increased in PTX- and DOX-treated cells in both the fibrous dense bipolar poles and radial arrays (Fig. 2B). The VCR-treated cells showed decrease in spindle length in radial array arrangements. A reduction in α-tubulin-presenting cell aggregation was observed after treatment with all chemotherapeutics (PTX, VCR, and DOX). Additionally, structural changes in nuclear DNA (blue) were observed, with more expansion in 12 µM 7HF treated-cells and minimal shrinkage in PTX- and DOX treated-cells after 24 h (Figs. 2A–2B)

To determine the effect of 7HF on spindle MT formation, α-, β-tubulin polymerization was examined in a cell-free system for 1 h (Fig. 2C and Spreadsheet S1). All concentrations of 7HF (6, 12, and 24 µM) affected tubulin formation. For all 7HF concentrations, tubulin nucleation occurred for 10 min, followed by tubulin growth till 40 min and tubulin equilibrium. After 10 min of nucleation, all 7HF concentrations triggered faster tubulin polymerization than the control. However, tubulin polymerization and relative fluorescence intensity was reduced in a 7HF concentration-dependent manner. Tubulin polymerization was relatively increased by DOX, and PTX and reduced by VCR treatments. PTX stimulated tubulin nucleation within 5 min (fastest among the three drugs), followed by tubulin growth till 40 min, while maximum suppression of tubulin formation was observed in case of VCR.

The expression of proteins involved in MT dynamics at the G2/M phase arrest was examined in 7HF-treated cells both with respect to 7HF concentration and time of incubation (Figs. 2D–2E). For assessing concentration dependence, the cells were incubated with 0, 6, 12, and 24 µM 7HF for 24 h (Fig. 2E and Fig. S2B). Bub3 was significantly upregulated in 6 µM 7HF-treated cells compared to that in the control. Similarly, upregulation of cyclin B1 and p-Cdk1 (Tyr15) was observed in concentration-independent and -dependent manners, respectively. For assessing time dependence, the cells were treated with 6 µM 7HF for 0, 12, 24, and 48 h (Fig. 2D and Fig. S2A). Bub3 was significantly upregulated between 12–24 h. Cyclin B1 was significantly upregulated at 12 h. In addition, p-Cdk1 (Tyr15) was significantly upregulated between 12 and 24 h and gradually downregulated at 48 h. All comparisons were made with the control group.

### Effect of 7HF on cell proliferation and expression of DDR proteins

**Figure 3:**
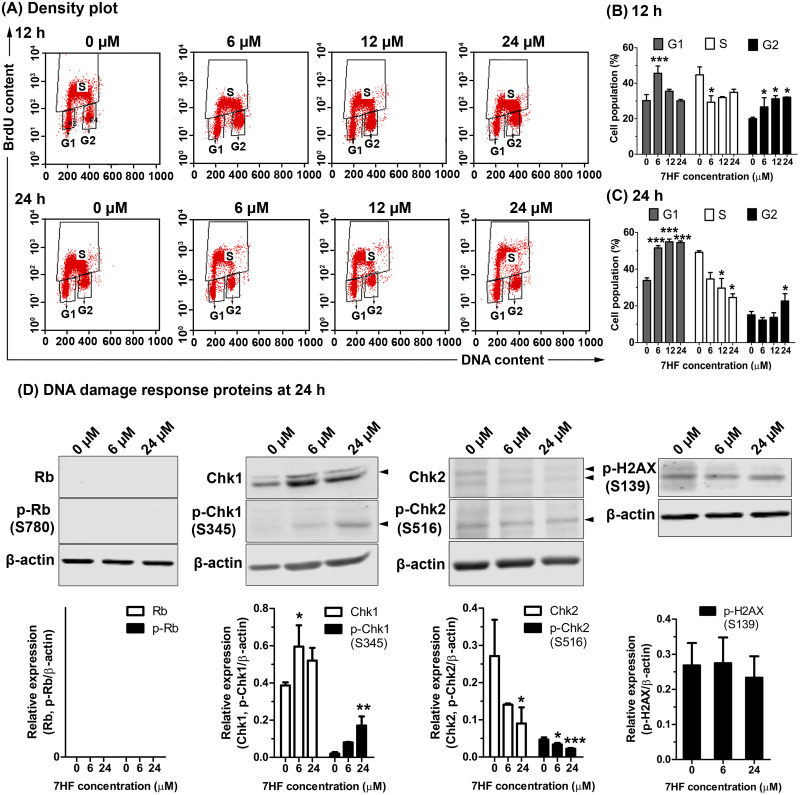
Effect of 7-α-hydroxyfrullanolide (7HF) on cell proliferation and expression of DNA damage response (DDR) proteins in MDA-MB-468 cells. (A) Density plots of cells treated with 0, 6, 12, and 24 µM 7HF for 12 h and 24 h. Left-lower, middle-top, and right-lower quadrants represent the number of cells in G1, S, and G2 phases, respectively. (B–C)The histograms represent the percentages of cells in G1, S, and G2 phases after 12 and 24 h treatment. (D) Expression of DDR proteins, including Rb, p-Rb (Ser780), Chk1, p-Chk1 (Ser345), Chk2, p-Chk2 (Ser516), and p-H2AX (Ser139), in cells treated with 6 and 24 µM 7HF for 24 h. S, serine; black arrowhead, an indicated protein band. Two or three independent experiments were performed. Mean difference was statistically analyzed using one-way ANOVA and Bonferroni’s (Figs. 3B–3C) and Dunnett’s (Fig. 3D) multiple comparison test. *p < 0.05, ***p < 0.001, compared to 0 µM (control).

The effect of MT dynamics on cell proliferation and DNA synthesis in 7HF-treated cells was determined using BrdU and PI staining, and FACS analysis. The cells were treated with various concentrations of 7HF (6, 12, and 24 µM) for 12 and 24 h (Figs. 3A–3C and Fig. S3); 7HF-concentration dependence analysis at 12 h (Figs. 3A–3B) revealed that the number of G1 phase cells was significantly higher at 6 µM (45.61%) than that of the control (30.23%). The number of S phase cells increased gradually in a concentration-dependent manner from 6 to 24 µM (29.32%–34.89%), but was lower than that of the control (44.76%). The number of cells in the G2 phase was significantly higher than that in the control (20.07%) and increased in a concentration-dependent manner at 6 µM (26.68%), 12 µM (31.24%), and 24 µM (32.14%). Concentration dependence analysis at 24 h (Figs. 3A and 3C) revealed that the number of cells in the G1 phase was significantly higher than that in the control (33.86%) and increased in a concentration-dependent manner from 6 to 24 µM (51.56–54.87%). The number of S phase cells was clearly lower than that in the control (49.19%) when treated with 6–24 µM 7HF (34.64–24.55%), although the number of cells treated with 24 µM 7HF (22.64%) was higher than that in the control (15.11%). The proportion of cells in the G2 phase appeared independent of time of treatment (12–24 h). In contrast, the proportion of cells in the G1 phase occurred in a time-dependent manner (Figs. 3B–3C). Hence, we concluded that 7HF triggers G2/M and G1 phase arrests in concentration- and time-dependent manner.

7HF treatment significantly increased the number of G1 phase cells at 24 h (Fig. 3C). DDR proteins such as Rb, p-Rb (Ser780), Chk1, p-Chk1 (Ser345), Chk2, p-Chk2 (Ser516), and p-H2AX (Ser139) were subsequently measured in the cells treated with 6 and 24 µM 7HF for 24 h, using western blotting (Fig. 3D and Fig. S4). However, the relative levels of Rb and p-Rb were not detected. Compared to that in the control, Chk1 and p-Chk1 were significantly upregulated, while Chk2 and p-Chk2 were significantly downregulated in a 7HF-concentration-dependent manner in the treated cells. p-H2AX expression was insignificantly differed in 7HF-treated group of 6 and 24 µM.

### Drug-likeness properties of 7HF

The Swiss ADME analysis of 7HF revealed its physicochemical properties, pharmacokinetics, drug-likeness, and medicinal properties. 7HF showed predicted drug-likeness, as it followed the Lipinski’s rule of Five (Lipinski et al., 2001; Yang & Hinner, 2015): MW 248.32, 3 H-bond acceptors, 1 H-bond donor, Clog P 2.46, and high gastrointestinal (GI) absorption.

### Predicted interaction of 7HF with α- and β-tubulins

**Figure 4:**
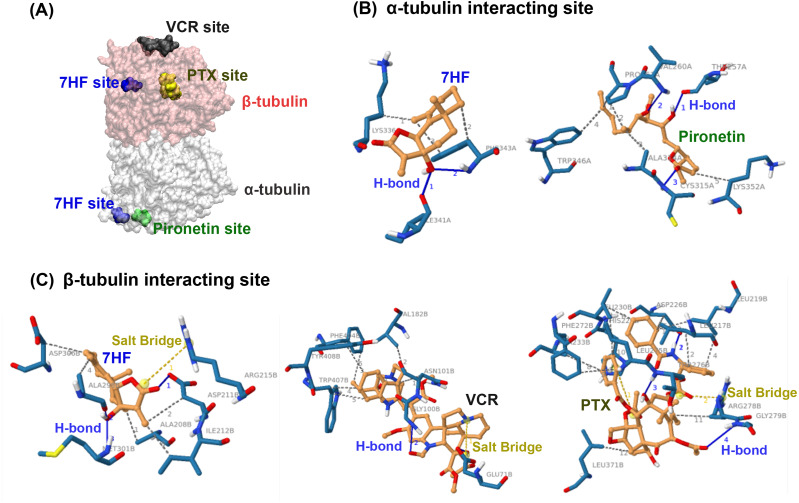
Predictive binding of 7-α-hydroxyfrullanolide (7HF) and the inhibitors with α- and β-tubulins. (A) Predictive binding locations of 7HF (blue), VCR (black), PTX (yellow), and pironetin (green) in the α/β-tubulin complex. The α- and β-tubulins are shown as grey and pink surface structures, respectively. (B) Interacting sites of ligands (orange): 7HF and pironetin with amino acids (navy blue) in α-tubulin structure are shown. (C) Interacting sites of ligands (orange): 7HF, PTX, and VCR with the amino acids (navy blue) in β-tubulin structure are shown. The blue, dotted grey, and dotted yellow lines depict H-bonds, hydrophobic interactions, and salt bridges, respectively. PTX, paclitaxel; VCR, vincristine.

Molecular docking was used to predict whether 7HF targeted α- or β-tubulins of MTs. 7HF and pironetin bind to α-tubulin with relative binding energies (Δ*G*_bind_) of −6.77 and −5.45 kcal/mol, respectively (Table 1). However, 7HF, PTX, and VCR bind to β-tubulin with Δ*G*_bind_ values of −7.12, −8.05, and −7.09 kcal/mol, respectively (Table 1). 7HF bound to both α- and β-tubulin sites, which was distinct from that observed with pironetin (a known α-tubulin inhibitor) and PTX and VCR (also known β-tubulin inhibitors), as shown in Fig. 4A. Table 1 and Figs. 4B–4C show 7HF-inhibitor interactions in terms of amino acid residues of various tubulin proteins. The initial binding site was at α-tubulin (Fig. 4B). A hydroxyl group (-OH) at C-7 in 7HF interacted with α-tubulin *via* hydrogen bonds (H-bond) with Ile341 and Phe343. An additional hydrophobic interaction with Lys336 and Phe343 further stabilized the 7HF binding. Compared to 7HF, pironetin formed H-bonds with Cys315, and backbone atoms of Thr257 and Val260. The neighboring amino acids such as Trp346, Ala314, and Lsy352 also supported the binding *via* hydrophobic interactions.

**Table 1:** Interaction of drugs with tubulins and their relative binding energies (ΔG_bind_).

**Compounds**	**ΔG_bind_** **(kcal/mol)**	**H-bonds**	**Salt bridges**	***π*–*π* stacking**
**α-tubulin**				
-7HF	−6.77	Ile341, Phe343	–	–
-Pironetin	−5.45	Thr257, Val260, Cys315	–	–
**β-tubulin**				
-7HF	−7.12	Asp211, Ala298, Met301	Arg215	–
-PTX	−8.05	Asp226, Thr276, Gly279	His229, Arg278	His229
-VCR	−7.09	Glu71, Gly100	Glu71	–

**Notes.**

7HF 7-α-Hydroxyfrullanolide PTX paclitaxel VCR vincristine

7HF also interacted with β-tubulin (Table 1 and Fig. 4C). A lactone ring established a salt bridge with Arg215. The C-12 ketone (C =O) formed H-bonds with Asp211. A hydroxyl (-OH) group at C-7 formed H-bonds with Ala298 and Met301. Some amino acids, such as Ala208, Ala298, Asp211, Asp306, and Ile212, exhibited hydrophobic interactions with 7HF. His229 and Arg278 carboxylic acids formed salt bridges to PTX. The PTX-protein complex displayed *π*–*π* stacking with His229. Furthermore, hydrophobic interactions with Leu217, Leu219, Leu230, Leu275, Leu 371, Asp226, Ala233, Phe272, and Arg278 contributed to PTX-β-tubulin binding. VCR-tubulin formed a salt bridge at Gln71 tetramine, and its H-bonds were formed by VCR. Amino acids such Asn101, Val182, Phe404, Trp407, and Tyr408 assisted in VCR binding *via* hydrophobic interactions.

## Discussion

A calibration curve of 7HF at 268 nm was correlated with increasing of concentration as reported previously (Rahman et al., 2016). The spectrophotometric results at 268 nm were used to examine chemical uptake in cancer cells. 7HF could pass through MDA-MB-468 cells as was apparent from the absence of 7HF in the medium at 268 nm. The passive penetration of the cancer cell membrane was also supported by Swiss ADME prediction based on Lipinski’s drug likeness (Daina, Michielin & Zoete, 2017; Lipinski et al., 2001). Swiss ADME predicted that 7HF preferably follows passive diffusion and possessed the high GI absorption property. These predicted features of 7HF are critical for designing and developing 7HF-based drugs.

TNBC cell lines can be used for developing better treatment options for patients with TNBC (Chavez, Garimella & Lipkowitz, 2010). The MDA-MB-468 cell line is a basal-like 1 subtype of TNBC that exhibits highly aggressive adenocarcinoma and harbors aberrations in numerous genes involved in cell division and DDR (Lehmann & Pietenpol, 2014; Yin et al., 2020). Hence, MDA-MB-468 cells may be a representative TNBC model that can be used to determine the responses of MTs and DDR pathways to 7HF treatment.

MT instability is critically involved in stress response of cancer cells (Parker, Kavallaris & McCarroll, 2014). Most cellular MTs exhibit cycles of growth, shortening, and regrowth (Gudimchuk & McIntosh, 2021). MT instability in MDA-MB-468 cells varied in 7HF concentration- and time-dependent manner (Fig. 2A). The density of the MTs in the spindle is high at the bipolar pole, and tubulin expression is the highest at the G2/M-phase transition (Sato & Toda, 2004; Syred et al., 2013). We observed that 7HF was internalized by cancer cells by 12 h, following which it disrupted MT dynamics, leading to G2/M arrest. 7HF induced G2/M phase arrest by upregulating Bub3, cyclin B1, and p-Cdk1 (Tyr15) (Chimplee et al., 2022), particularly at 6 µM concentration and after incubation of 12–24 h (Figs. 2D–2E). Bub3 and BubR1 are ubiquitously expressed throughout the cell cycle; however, Bub3 and BubR1 production was high in the G2/M phase (Liu & Zhang, 2016; Sudakin, Chan & Yen, 2001). Bub3-BuR1 and Cdc20-Mad2 are evolutionarily conserved mitotic checkpoint proteins that form a mitotic checkpoint complex (MCC) only at mitosis (Han et al., 2014; Liu & Zhang, 2016). The active MCC is an effector of the anaphase-promoting complex/cyclosome (APC/C, an E3 ubiquitin ligase). APC/C bound to the MCC prevents cyclin B1 and securin degradation during spindle assembly checkpoint and the detachment of sister chromatids from the kinetochore (Liu & Zhang, 2016; Matthews, Bertoli & De Bruin, 2021). In general, cyclin B1 expression is temporally restricted to the G2 phase and early mitosis transition in human cells (Lindqvist, Rodriguez-Bravo & Medema, 2009). Mad2 is also found in 7HF-treated cells (Chimplee et al., 2022). Thus, 7HF might increase Bub3-Mad2 (MCC) formation and sustain cyclin B1 levels to check MT spindle formation during G2/M arrest. Cdk1 is inhibited when phosphorylated by Wee1 at Tyr15 (Schmidt et al., 2017). Phosphorylated Cdk1-cyclin B1 complex is inactive and blocks entry into mitosis (Schmidt et al., 2017). Indeed, high levels of p-Cdk1 (Tyr15) have been shown to be associated with G2/M phase arrest following DNA damage in different cancer cell types (Ghelli Luserna di Rorà et al., 2020). 7HF might result in accumulation of cyclin B1-p-Cdk1. In fact, cells in the G2 phase generally correct errors generated during DNA replication and prepare proteins before rapid growth and mitosis (Jackman, 2011; Kousholt, Menzel & Sorensen, 2012).

MT dynamics is a crucial factor regulating DNA damage repair during cell division; perturbations in MT dynamics lead to genomic instability (Lottersberger et al., 2015; Petsalaki & Zachos, 2020). Arrest in the G2/G1 phases after 12 h of 7HF treatment could be due to MT instability and persistence of DNA aberrations (Figs. 2A and 3A–3B). Variations in the anti-mitotic activity and disruption of MT dynamics in numerous cancer cell types have been observed due to differences in the chemical carbocyclic structure of SLs, all of which trigger G2/M arrest *via* the upregulation of p-Cdk1 (Try 15) and cyclin B1 (Bosco & Golsteyn, 2017). Santamarine (Ma et al., 2009), parthenolide (Whipple et al., 2013), and costunolide (Bocca et al., 2004; Liu et al., 2011a; Whipple et al., 2013) are eudesmanolides (6/6 bicyclic SLs) that are similar to 7HF. The mode of action of eudesmane SLs involves perturbation of MT stability at the levels of both tubulin polymerization and depolymerization, and upregulation of p-Cdk1 (Tyr15) and cyclin B1 in leukemia (CCRF-CEM murine), breast cancer (MCF-7), and hepatoma (HA22T/VGH) cells. 6-O-angeloylplenolin, coronopilin, pulchelloid A, and hymenoratin, 5/7-bicyclic pseudoguaianolide SLs also induce high p-Cdk1 and/or Cdk1 and cyclin B1 expression in certain cancer cell types. (Bosco et al., 2021; Cotugno et al., 2012; Liu et al., 2011b; Molina et al., 2021). However, the role of Bub3 and its partners in the modulation of MCC after SL treatment is not clear (Steinbruck, Pereira & Efferth, 2010).

The upregulation of p-Cdk1 (Tyr15) appears to maintain cyclin B1 accumulation with G2/M arrest, followed by DNA damage, after 24 h of treatment with 7HF. The G1 phase arrest was characterized by low MT dynamics, with the lengthening of the radial array in 7HF-treated cells at 24 h (Figs. 2A, 3A–3C) (Sato & Toda, 2004; Syred et al., 2013). MTs recruit trafficking proteins to repair aberrant DNA, leading to G1 phase arrest (Lottersberger et al., 2015). Previous studies have shown that 7HF inhibits DNA Top I activity; 7HF may affect pre-replicative stress or single-stranded DNA break in the G1 phase arrest by upregulating Chk1 *via* Ataxia-Telangectasia and Rad3 related (ATR) kinase signaling (Champoux, 2001; Ju et al., 2020; Petsalaki & Zachos, 2020; Uppatanpreecha, 2009). Chk1 is a key downstream regulator of ATR response and is phosphorylated by ATR at Ser345 (Ju et al., 2020; Petsalaki & Zachos, 2020). Phosphorylation of Chk1 at Ser345 is essential for its biological activity (Petsalaki & Zachos, 2020). In 7HF-treated cells, Chk1, p-Chk1 (Ser345), p-Cdk1 (Tyr15), and cyclin B1 levels increased after 24 h (Figs. 2D–2E and 3D). Chk1 plays key roles in both late G1-S phase (to inhibit DNA replication initiation) and G2-M-phase checkpoints (to check MT spindle assembly) (Ju et al., 2020; Petsalaki & Zachos, 2020). Active Chk1 (p-Chk1 Ser345) dissociates from chromatin and phosphorylates and inactivates the Cdc25 family phosphatases (Petsalaki & Zachos, 2020). Chk1 stimulates Wee1 kinase to phosphorylate Cdk1 at Tyr15 (Cdk1-cyclin B1 inhibition). High Chk1-p-Chk1-Wee1-p-Cdc25-p-Cdk1-cyclin B1 activity prevents the onset of mitosis by activating the spindle assembly checkpoint, which persists till DNA damage is not repaired (Petsalaki & Zachos, 2020). A recent study has reported that DNA damage activates MT dynamics and is associated with the spindle checkpoint in the G1 phase arrest (Ma et al., 2021). SLs induce G2/M-G1 phase arrest *via* the proposed Chk1, p-Chk1-Cdc25s-p-Cdk1 (Tyr15)-cyclin B1 pathway. Costunolide upregulates Chk2 (Thr68), p-Cdc25c (Ser216), p-Cdk1 (Tyr15), and cyclin B1 in liver cancer (HA22T/VGH) cells (Liu et al., 2011a). Galiellalactone increases ATM/ATR, p-Chk1 (Ser345), and cyclin B1 protein levels in prostate cancer cells (Garcia et al., 2016). Coronopilin increases cyclin B1 and p-Cdk1 (Tyr15) levels; however, the upregulation of *γ*H2AX (p-H2AX Ser139) is involved in G1 phase arrest in leukemia (Jurkat and U937) cells (Cotugno et al., 2012).

In the present study, 7HF triggered G1 phase arrest independent of DDR *via* Rb, p-Rb, Chk2, p-Chk2, and *γ*H2AX signaling. Not surprisingly, Rb and p-Rb (Ser780) expression could not be detected in the 7HF-treated cells, as MDA-MB-468 cells are Rb-null lines (Robinson et al., 2013). However, the deficiency of Rb in breast cancer cells renders them highly sensitive to chemotherapy (DOX and methotrexate) and radiotherapy (Robinson et al., 2013). Reduction in the levels of Chk2, p-Chk2 (Ser516), and phosphorylated H2AX (p-H2AX, *γ*H2AX) was observed in the 7HF-treated cells at 24 h. 7HF might not induce DDR *via* dsDNA breaks in the G1/S/intra S phase checkpoints (Sharma, Singh & Almasan, 2012; Zannini, Delia & Buscemi, 2014).

The fibrous nature of the spindle increased in 7HF-treated cells was similar to that observed in 7HF-untreated cells and DOX-treated cells at 24 h (Figs. 2A–2B). PTX- and VCR-treated cells exhibited spindle lengthening at the bipolar pole and shrinkage of the radial spindle array, respectively (Fig. 2B). 7HF lowered spindle MT stability and α-, β-tubulin polymerization more than PTX (MT stabilizing agent) and VCR (MT destabilizing agent). 7HF bound to a pocket in β-tubulin that differed from the PTX and VCR sites (Fig. 4A). Two PTX-tubulin interaction sites have been previously reported (Field Jessica, Díaz José & Miller John, 2013). The initial site was located in luminal β-tubulin at position of S7 end (Phe 272), H1 (Asp226), H6-H7 (Leu217, Leu219, His 229, Leu 230, Ala 233), and M-loop (Leu275, Arg278). One of these two is the external pore type I site. An increase in lateral protofilament connections due to PTX and M-loop interaction in the luminal region is critical for tubulin stability. Additional amino acids (Phe214, Thr220, Thr221, and Pro222) occupy the pore type I site (Field Jessica, Díaz José & Miller John, 2013). This cavity is formed by the Lys216, Thr218, and Gly271 residues of the β1 subunit and the Arg77, Pro87, Asp88, and Phe90 residues of the β2 subunit (Barasoain et al., 2010).

The vinca alkaloid interacts with β-tubulin at 177-215 peptides (Mitra & Sept, 2004; Rai & Wolff, 1996). Vinorelbine, a vinblastine analogue, also interacts with Tyr224’s side chain (Lobert et al., 2000). The other side chain of Asp179 moves from nucleotide to drug (Gigant et al., 2005). Table 1 depicts a VCR tubulin interaction site. VCR structure prevented protein binding. Small ligand diffusion into tubulin was proposed here (Field Jessica, Díaz José & Miller John, 2013). Unlike PTX/VCR-tubulin interaction, the 7HF-tubulin complex formed hydrophobic and salt bridge contacts with Ile212 and Arg215, close to the external pore type I of MTs (Field Jessica, Díaz José & Miller John, 2013). It was found that 7HF gave Δ*G*_bind_ of −6.77 and −7.12 kcal/mol toward the exterior α- and β-tubulin sites (Field Jessica, Díaz José & Miller John, 2013). These numbers were not enough to clearly justify the preference. In addition, 7HF did not interact with any amino acid in luminal areas. Furthermore, 7HF formed H-bonds and hydrophobic interactions with Met301 (near Arg308 in LAU/PEL site) and Ala298, similar to natural MT stabilizing agents laulimalide (LAU) and peloruside A (PEL A) (Field Jessica, Díaz José & Miller John, 2013). Chemical bonds were formed between the 7HF lactone ring, ketone, and hydroxyl moieties and amino acid residues (Table 1 and Figs. 4B–4C). These moieties may contribute to a preference for 7HF-tubulin interactions (Ghantous et al., 2010).

As ligand- α-tubulin targeting is unknown, we predicted the molecular interaction in the 7HF- α-tubulin complex (Steinmetz & Prota, 2018). The 7HF-α-tubulin site differed from that of pironetin. A study showed that Cys316 and Lys352 of α-tubulin covalently bind to pironetin *via* Michael addition reactions (Prota et al., 2016; Usui et al., 2004; Yang et al., 2016). The binding was perfect and perturbed a major loop and helix of α-tubulin, inhibiting MT formation (Prota et al., 2016). Furthermore, the covalent bond promoted adducted pironetin formation and shifted the MT equilibrium (Bañuelos Hernández et al., 2014; Coulup & Georg, 2019; Steinmetz & Prota, 2018). Unlike the pironetin-α-tubulin interacting site, 7HF (-OH, C-7) used H-bonds at Ile341 and Phe343, as well as hydrophobic interactions at Lys336 and Phe343, in addition to the interactions with targeted Cys316 and Lys352. As previously stated, the results were insufficient to determine the specificity of 7HF with either α- or β-tubulin because 7HF was predicted to interact with both tubulin sites with close Δ*G*_bind_, falling within a range of 1 kcal/mol. Furthermore, the small size of 7HF may be the key for the molecular to enter the tubulin binding pocket. Finally, the specificity of 7HF remained a topic for further investigation.

## Conclusions

To the best of our best knowledge, this is the first report to investigate MT dynamics and its interaction with 7HF in TNBC cells, with the aim of deciphering the mechanism of action of 7HF in TNBC cells. Our findings revealed that 7HF entered the MDA-MB-468 cells, where it disrupted MT dynamics, along with DDR, *via* the induction of G2/M-G1 phase arrest, and upregulation of Chk1, p-Chk1 (Ser345), Bub3, p-Cdk1 (Tyr15), and cyclin B1. *In silico* analysis, 7HF preferably targets β-tubulin over α-tubulin, with the formation of H-bonds and salt bridges between 7HF moieties (lactone, ketone, and hydroxyl groups) and tubulin, which support the formation of the 7HF-MT complex. 7HF uptake prediction follows high passive diffusion and GI absorption routes. Hence, the results of this study will provide information for drug design, development, and therapy for TNBC patients.

## Supplemental Information

10.7717/peerj.13508/supp-1 Supplemental Information 1 Raw data of Figures 1–3 Absorbance values, original blots, and cell population for FACS analysis of three independent experiments.

10.7717/peerj.13508/supp-2 Supplemental Information 2 Raw data of maximum absorbance

10.7717/peerj.13508/supp-3 Supplemental Information 3 Raw data of absorbance of calibration curve

10.7717/peerj.13508/supp-4 Supplemental Information 4 Raw absorbance for 7HF uptake profile

10.7717/peerj.13508/supp-5 Supplemental Information 5 Raw data of cell cycle protein expression

10.7717/peerj.13508/supp-6 Supplemental Information 6 Raw data of FACS analysis

10.7717/peerj.13508/supp-7 Supplemental Information 7 Raw data of DDR protein expression

10.7717/peerj.13508/supp-8 Supplemental Information 8 Raw data of absorbance of tubulin polymerization assay

## Additional Information and Declarations

### Data Availability

The following information was supplied regarding data availability: The raw data are available in the Supplementary Files.
