# Supplementary material for: Anticancer mechanism of 7-α-hydroxyfrullanolide on microtubules and computational prediction of its target binding in triple-negative breast cancer cells
Source: PeerJ. 2022 May 27;10:e13508. doi: 10.7717/peerj.13508 (PMC9150694; doi:10.7717/peerj.13508)
Supplement: Supplemental Information 1 — Absorbance values, original blots, and cell population for FACS analysis of three independent experiments. [file peerj-10-13508-s001.pdf]

Anticancer Mechanism of 7-α-Hydroxyfrullanolide on Microtubules and Computational Prediction of its Target Binding in Triple-Negative Breast Cancer Cells

Siriphorn Chimplee[1], Carl Smythe[2], Varomyalin Tipmanee[1], Suchada Sukrong[3], Kanyanatt Kanokwiroon[1,*]

[1] Department of Biomedical Sciences and Biomedical Engineering, Faculty of Medicine, Prince of Songkla University, Hat Yai, Songkhla, Thailand
[2] School of Biosciences, University of Sheffield, Sheffield, United Kingdom
[3] Center of Excellence in DNA Barcoding of Thai Medicinal Plants, Department of Pharmacognosy and Pharmaceutical Botany, Faculty of Pharmaceutical Sciences, Chulalongkorn University, Bangkok, Thailand

Corresponding Author:
Kanyanatt Kanokwiroon[1]
15 Kanjanavanich, Khohong, Hat Yai, Songkhla, 90110, Thailand
Email address: kanyanatt.k@psu.ac.th

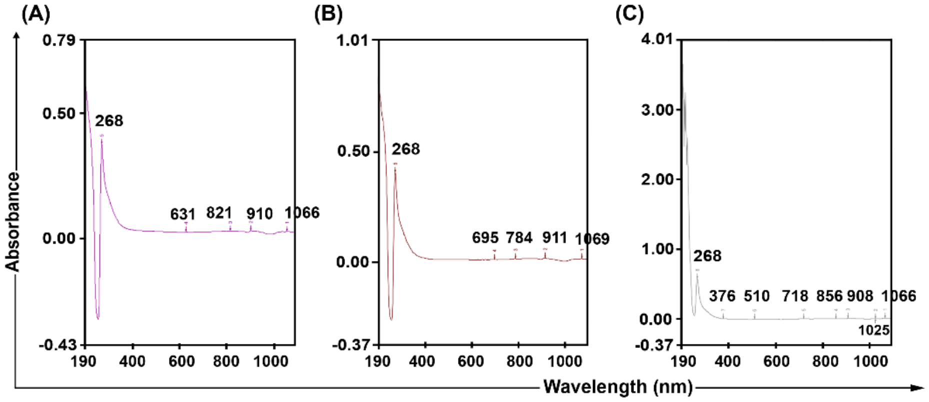

Figure S1: Maximum absorbance (λ_{max}) identification at 1,006.77 µM 7HF
(**A-C**) The peaks were identified in 1,006.77 µM of sample n1, n2, and n3, respectively

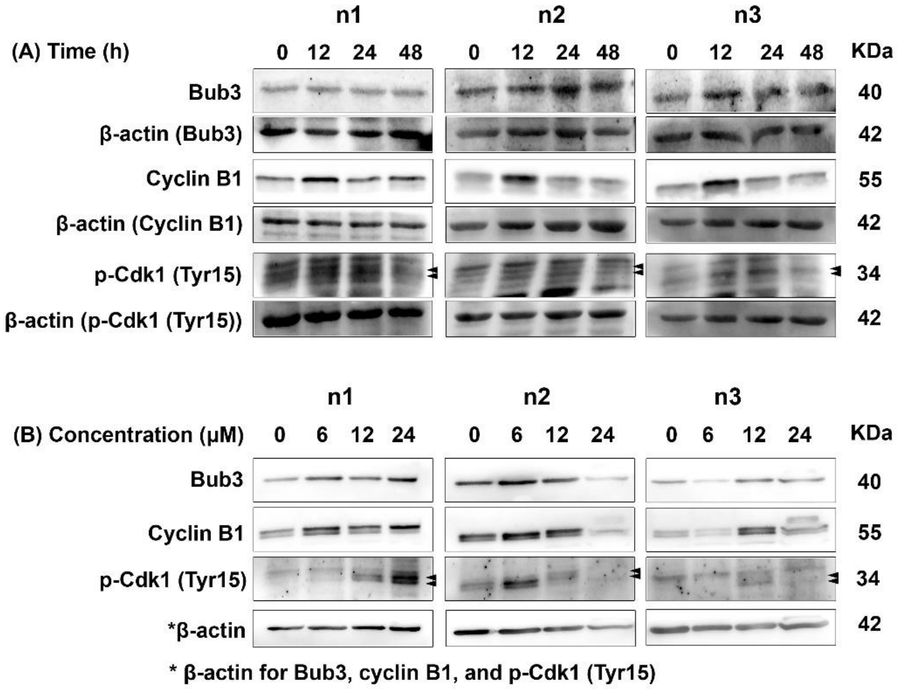

Figure S2: Protein expressions were evaluated using western blotting. (**A**) Bub3, cyclin B1, and p-Cdk1 (Tyr15) were detected at 6 µM 7HF for 0, 12, 24, and 48 h. (**B**) Bub3, cyclin B1, and p-Cdk1 (Tyr15) were performed at 0, 6, 12, and 24 µM 7HF for 24 h. These proteins were depicted along with their β-actin. The results were performed in two-three independent experiments (n1-n2/n1-n3). The experiment n1-n3 used for calculation of band intensity (or relative expression) from indicated protein and β-actin as representing in histogram Figure 2D-E. The full original western blot images in Figure 2D and Figure 2E were showed in page 3-5 and page 6-8 as follows: Bub3, Cyclin B1, and p-Cdk1 (Tyr15). In full original blot, 0, 6, 12, and 24 µM; 7HF treatment does (0, 6, 12, and 24 µM), 0, 12, 24, and 48 h; 7HF treatment times (0, 12, 24, and 48 h), Black outer square; border of full images, Red inner square and black head arrow; indicated protein bands, #; The blots are shown the same in Figure 2D-E.

Bub3 (40 KDa) and β-actin (42 KDa)

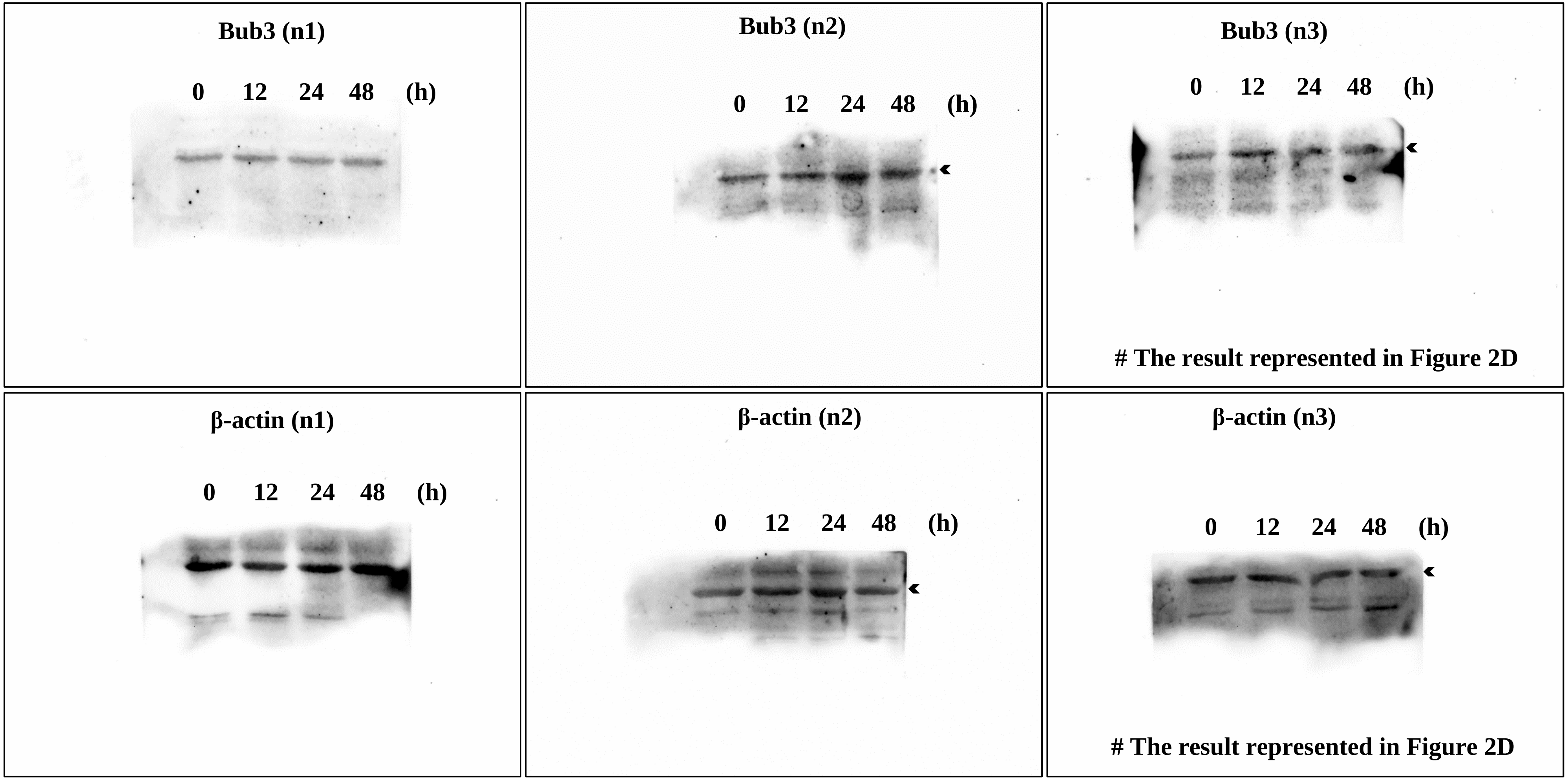

Cyclin B1 (55 KDa) and β-actin (42 KDa)

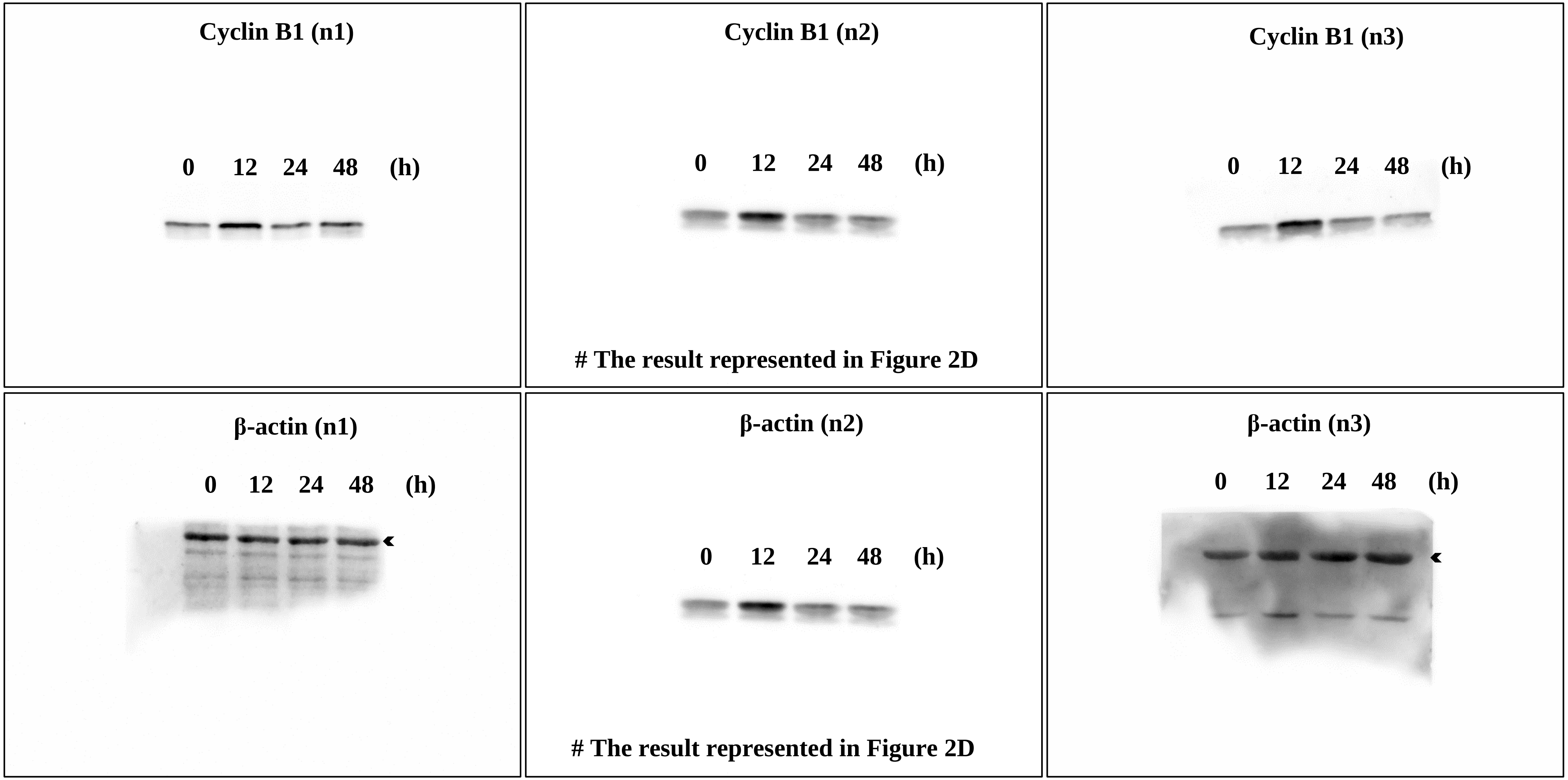

p-Cdk1 (Tyr15) (34 KDa) and β-actin (42 KDa)

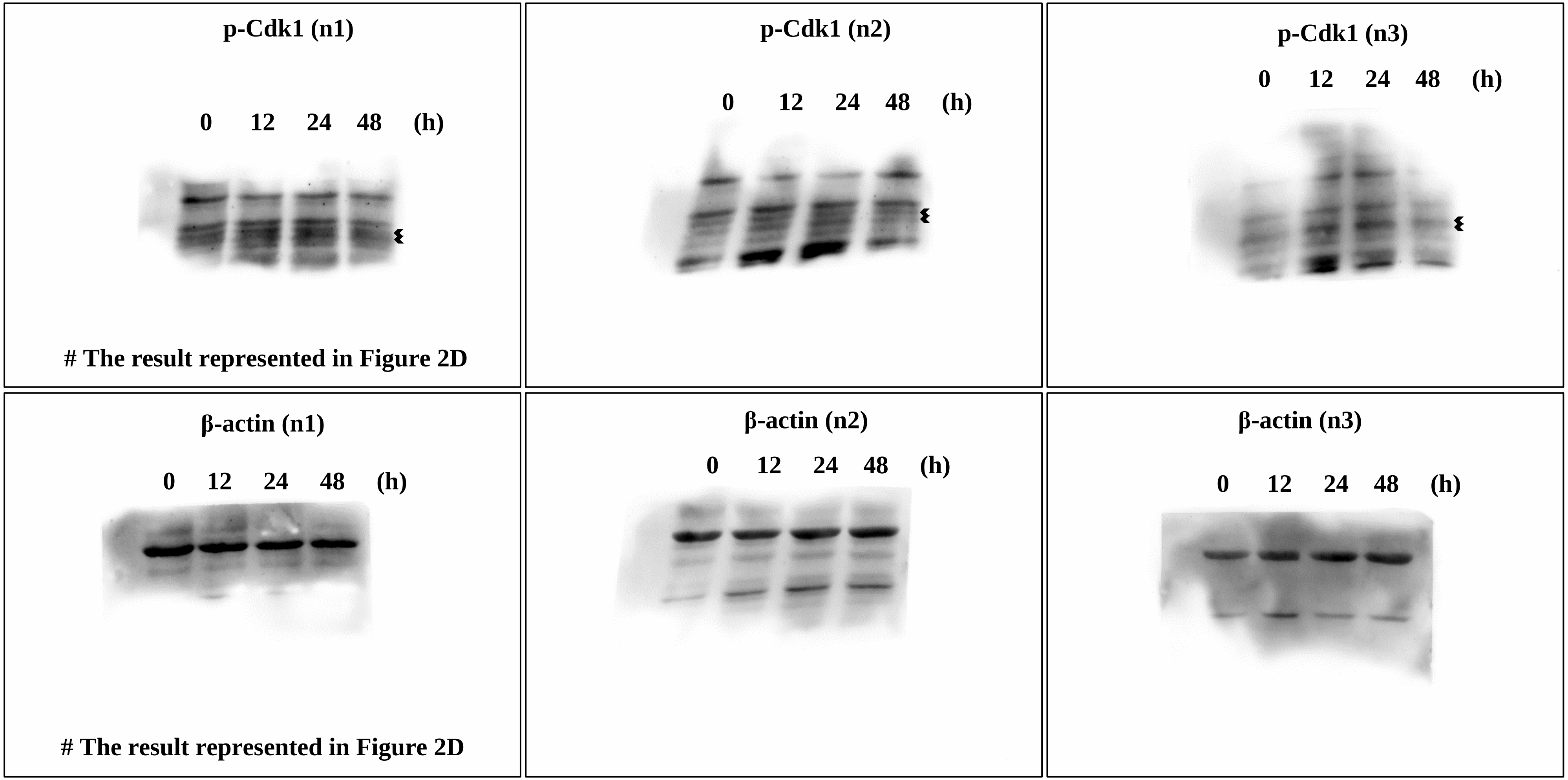

Bub3 (40 KDa) and β-actin (42 KDa)

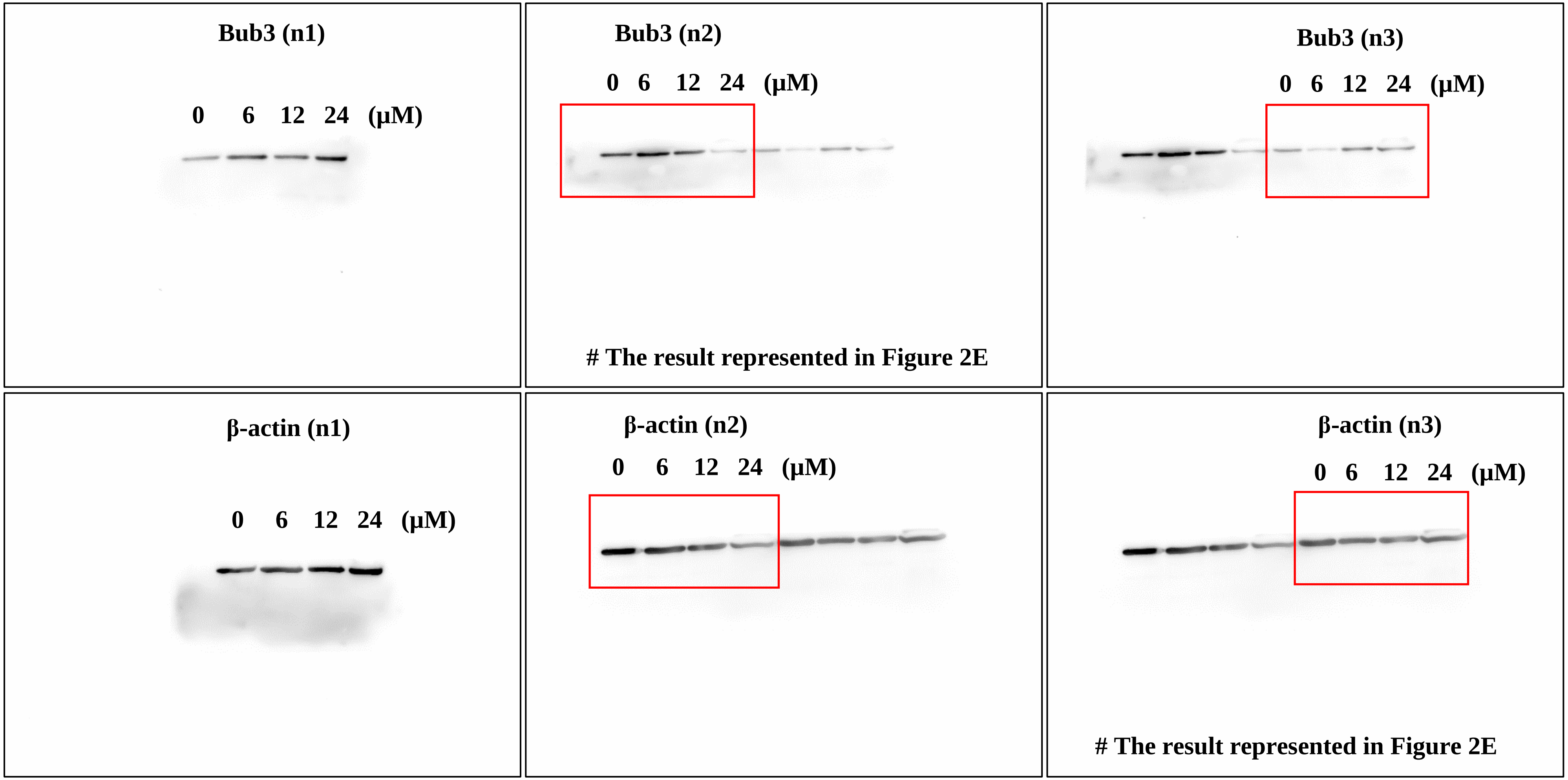

Note: Bub3 at 6 µM was used intensity calculation for n1-n2

Cyclin B1 (55 KDa) and β-actin (42 KDa)

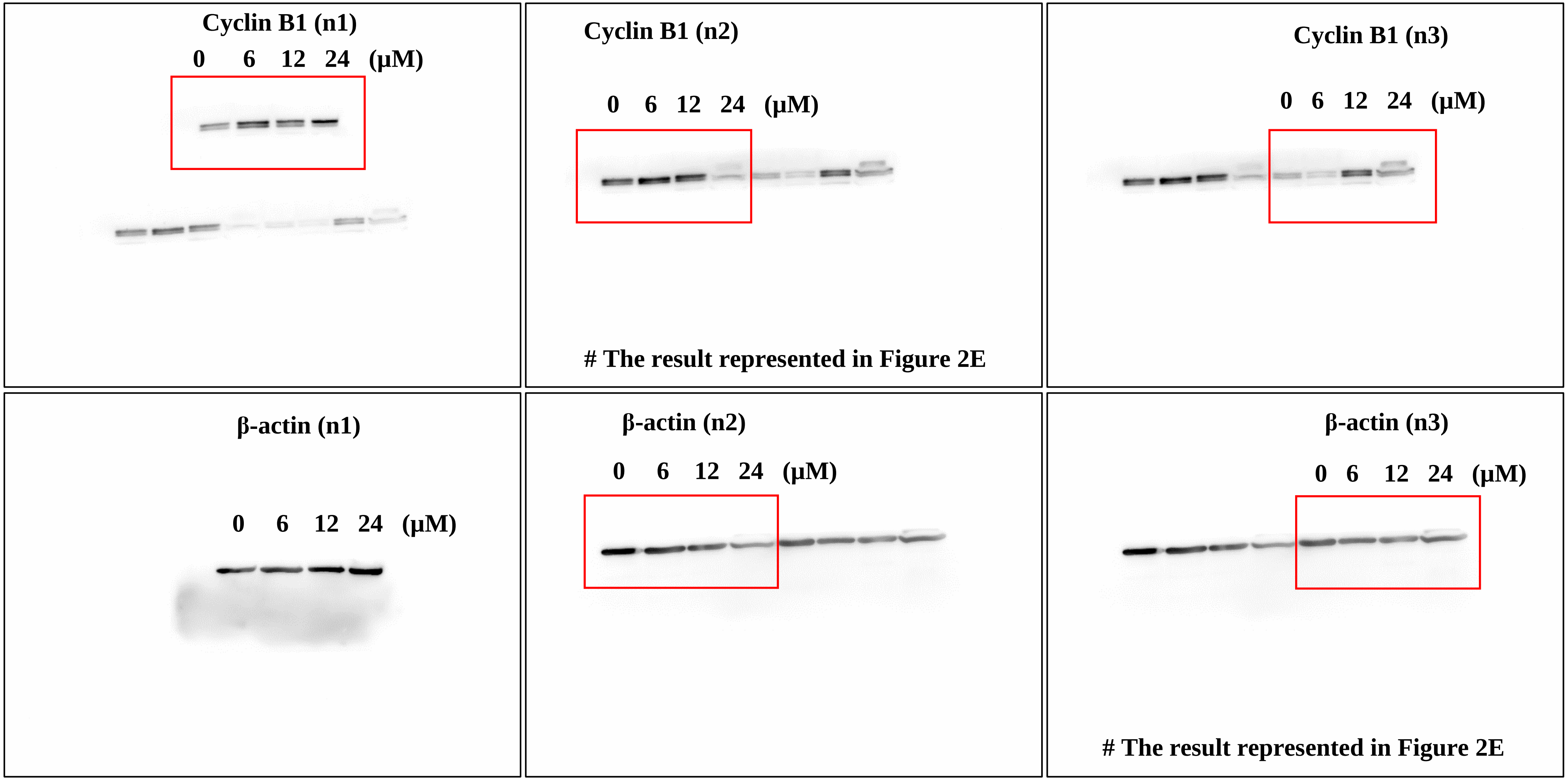

Note: Cyclin B1 at 6 μM was used intensity calculation for n1-n2

p-Cdk1 (Tyr15) (34 KDa) and β-actin (42 KDa)

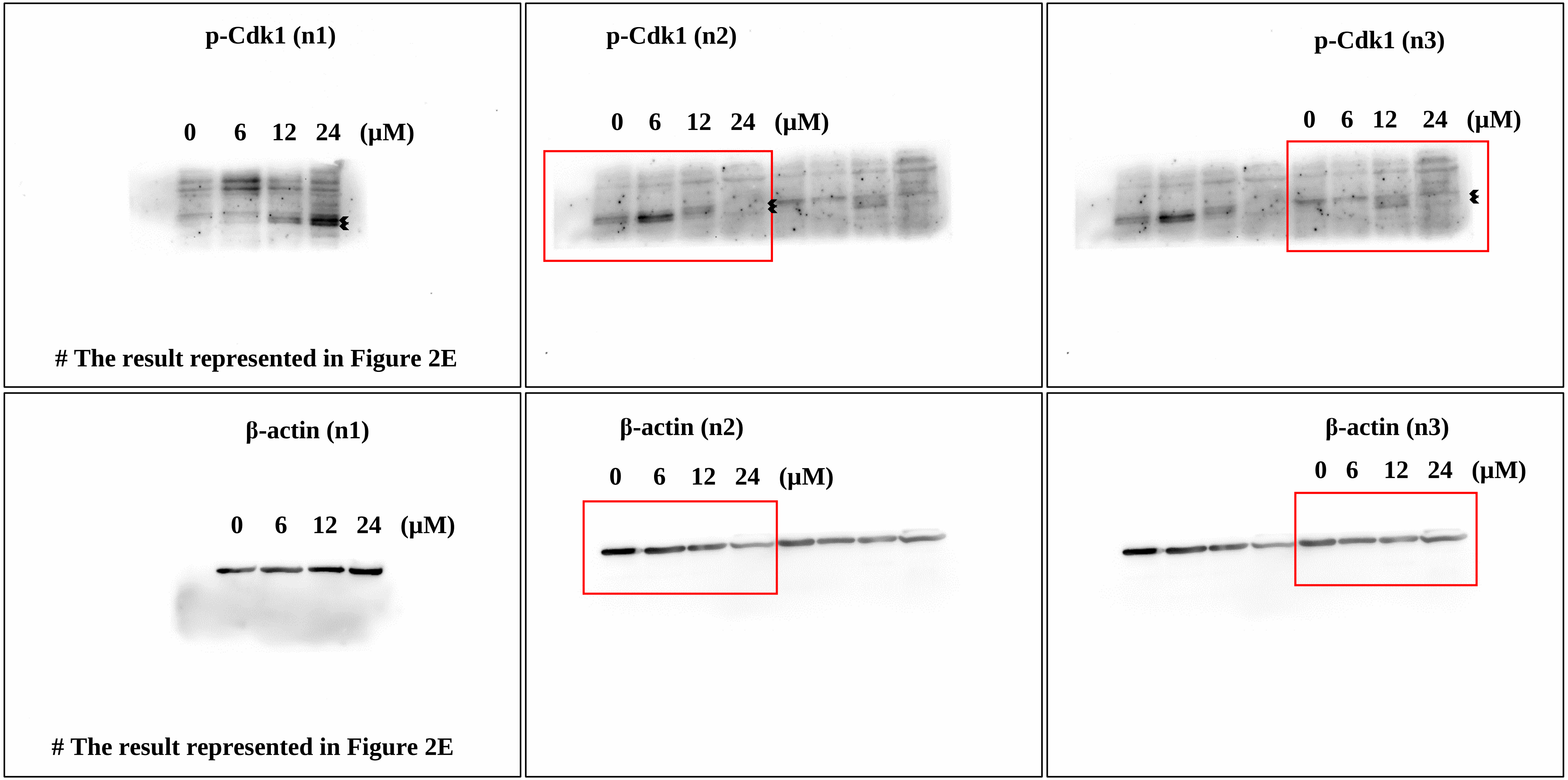

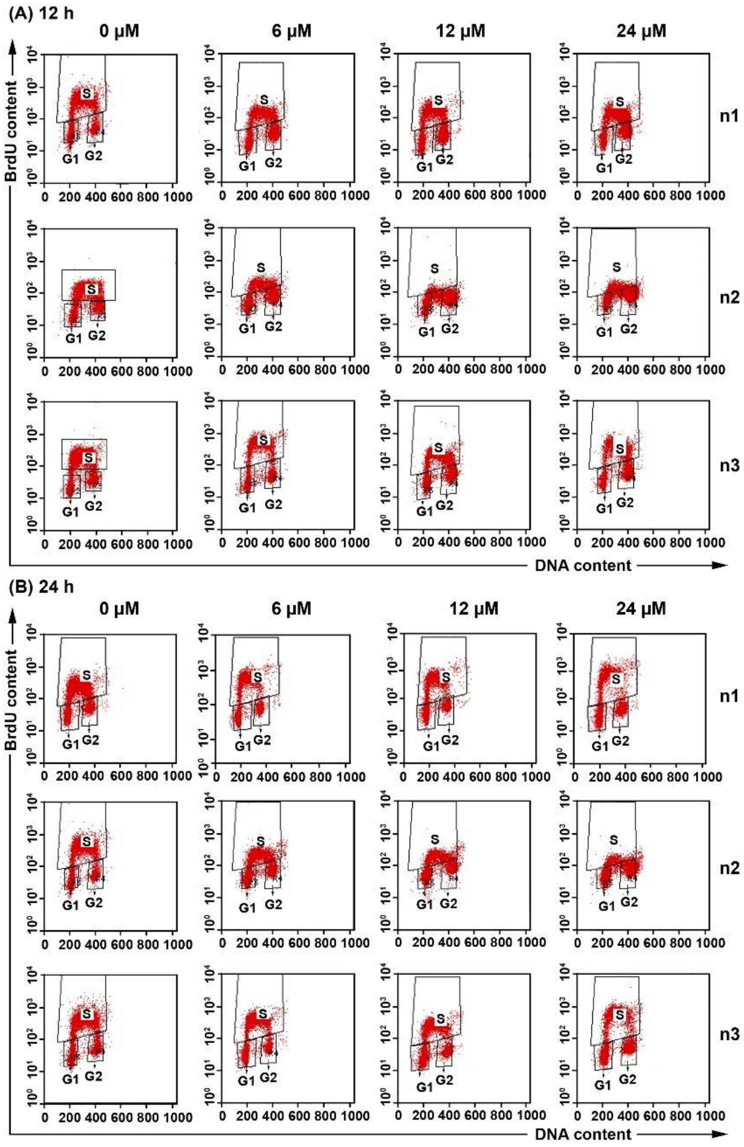

Figure S3: Cell population was evaluated bromodeoxyuridine plus propidium iodide staining using fluorescence assisted cell sorting analysis in 7HF-treated cells at 0, 6, 12, and 24 µM for 12 h (**A**) and 24 h (**B**). Left-lower, middle-top, and right-lower quadrants represent the number of cells in G1, S, and G2 phases, respectively. BrdU; bromodeoxyuridine, n1-n3; the sample 1, 2, and 3.

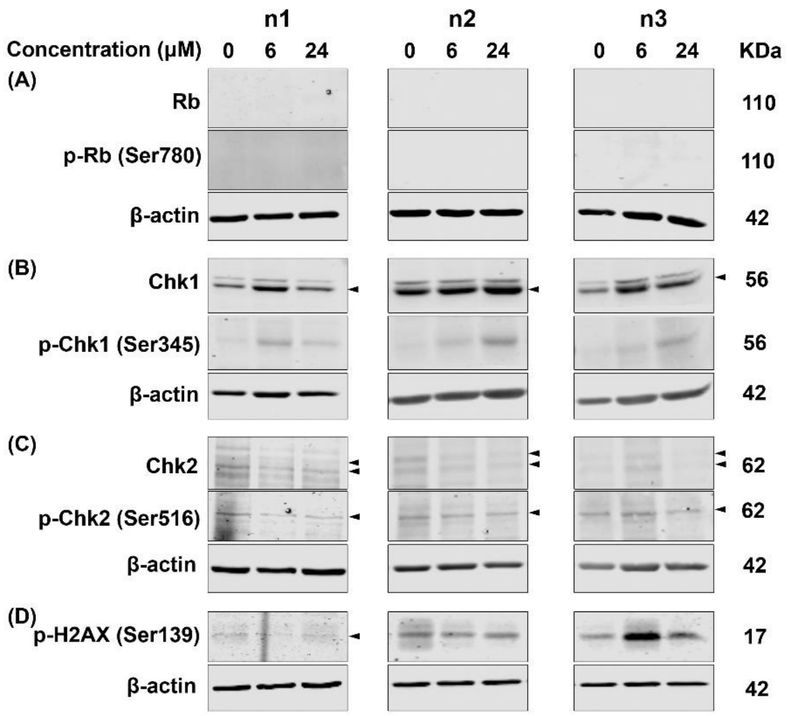

Figure S4: Protein expressions were evaluated using western blotting. (**A**) Rb and p-Rb Ser780, (**B**) Chk1 and p-Chk1 Ser345, (**C**) Chk2 and p-Chk2 Ser516, and (**D**) p-H2AX Ser139 were examined at 6 and 24 µM 7HF for 24 h. These proteins were depicted along with their β-actin. The results were performed in two-three independent experiments (n1-n2/n1-n3). The experiment n1-n3 used for calculation of band intensity (or relative expression) from indicated protein and β-actin as representing in histogram Figure 3D. The full original western blot images in Figure 3D were showed in page 11-17 as follows: Rb, p-Rb Ser780, Chk1, p-Chk1 Ser345, Chk2, p-Chk2 Ser516, and p-H2AX Ser139. In full original blot, 0, 6, and 24 µM; 7HF treatment does (0, 6 and 24 µM), Black outer square; border of full images, Red inner square and black head arrow; indicated protein bands, # and arrow; The blots are shown the same in Figure 3D. Note. Original full fluorescence western blot images were also depicted in page 18-21 as follows: Rb/p-Rb, Chk1/p-Chk1, Chk2/p-Chk2, and p-H2AX. β-actin was along with the proteins. M; Protein marker, White square and head arrow; indicated protein bands.

Rb (110 KDa) and p-Rb Ser780 (110 KDa)

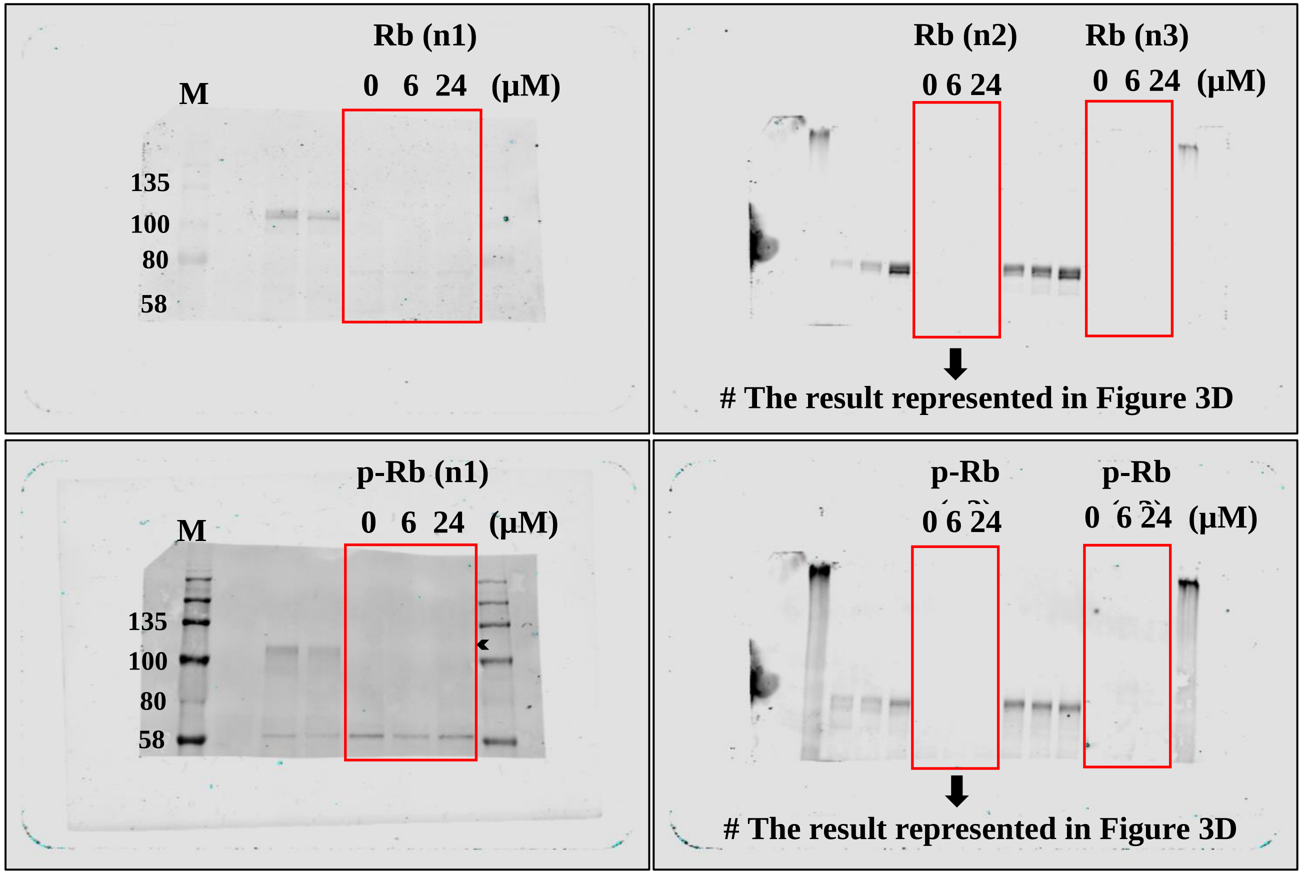

β-actin (42 KDa) of Rb and p-Rb

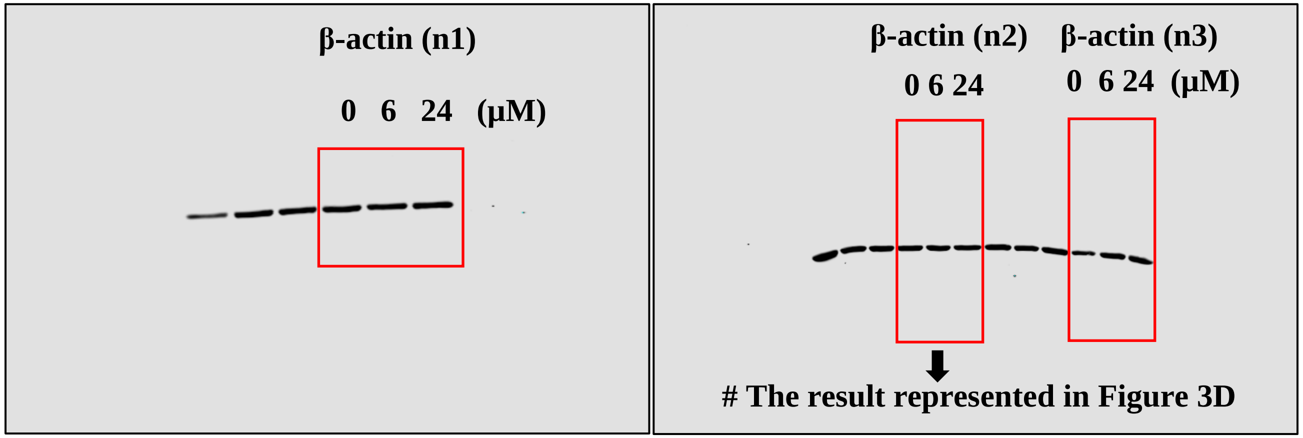

Chk1 (56 KDa) and p-Chk1 Ser345 (56 KDa)

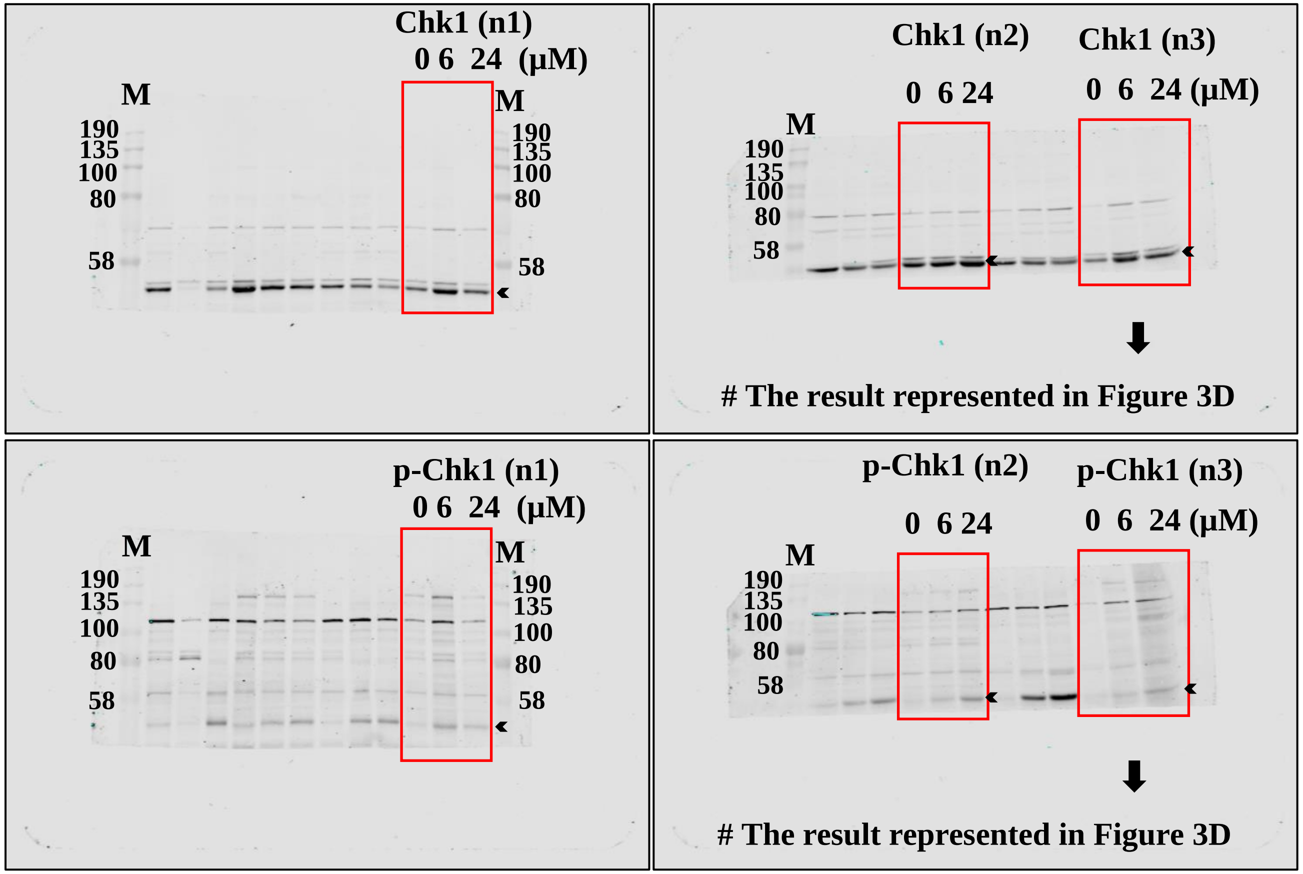

β-actin (42 KDa) of Chk1 and p-Chk1

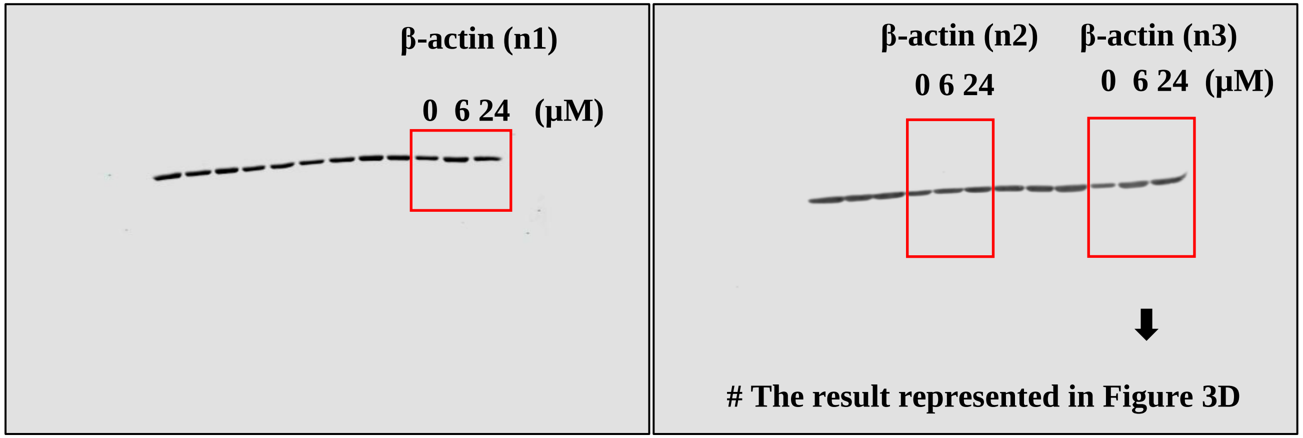

Chk2 (62 KDa) and p-Chk2 Ser516 (62 KDa)

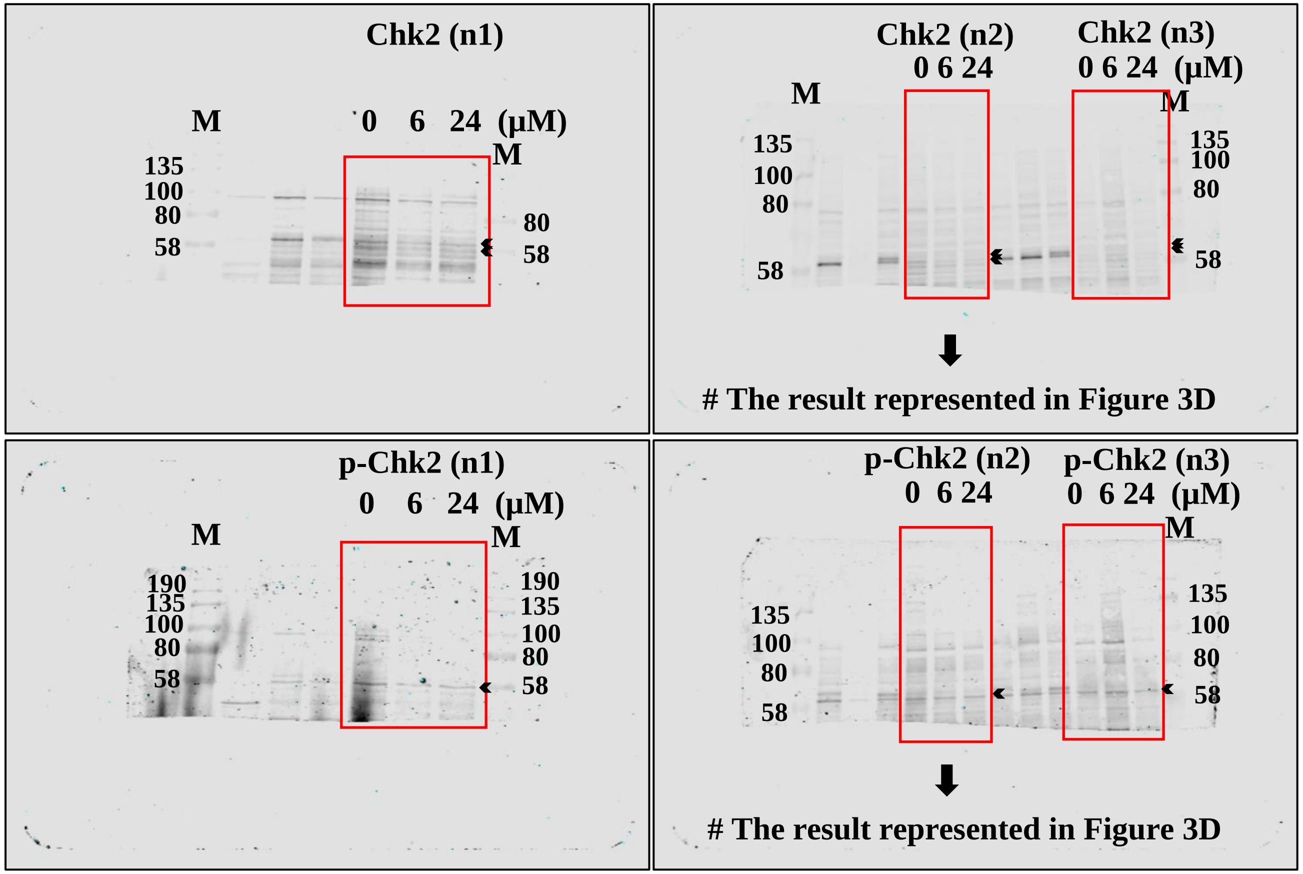

β-actin (42 KDa) of Chk2 and p-Chk2

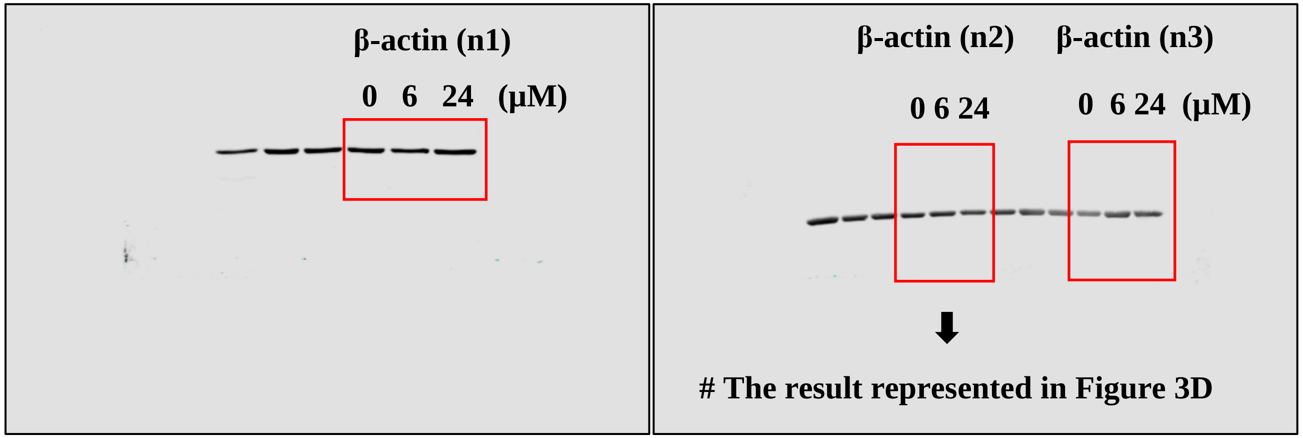

p-H2AX Ser139 (17 KDa)

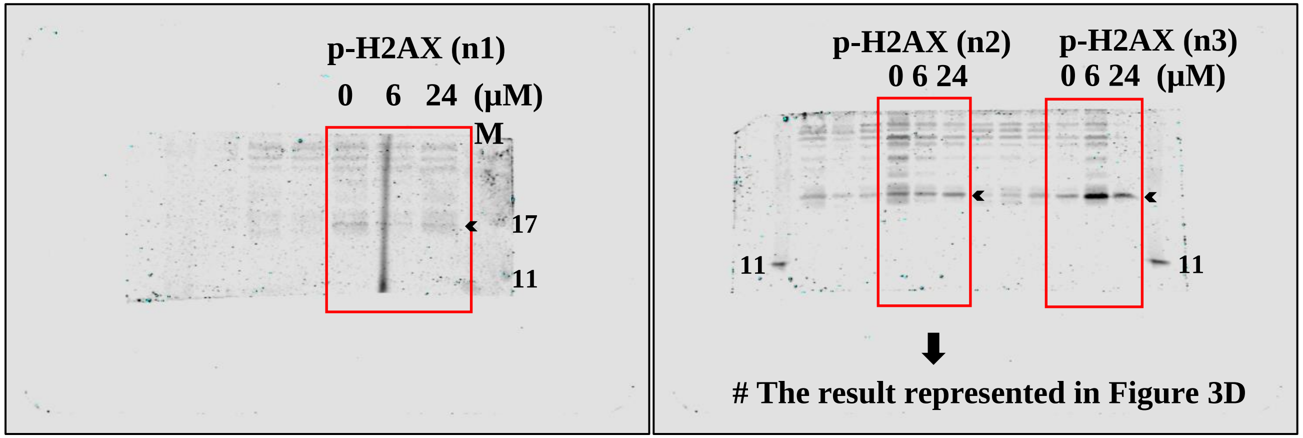

β-actin (42 KDa) of p-H2AX

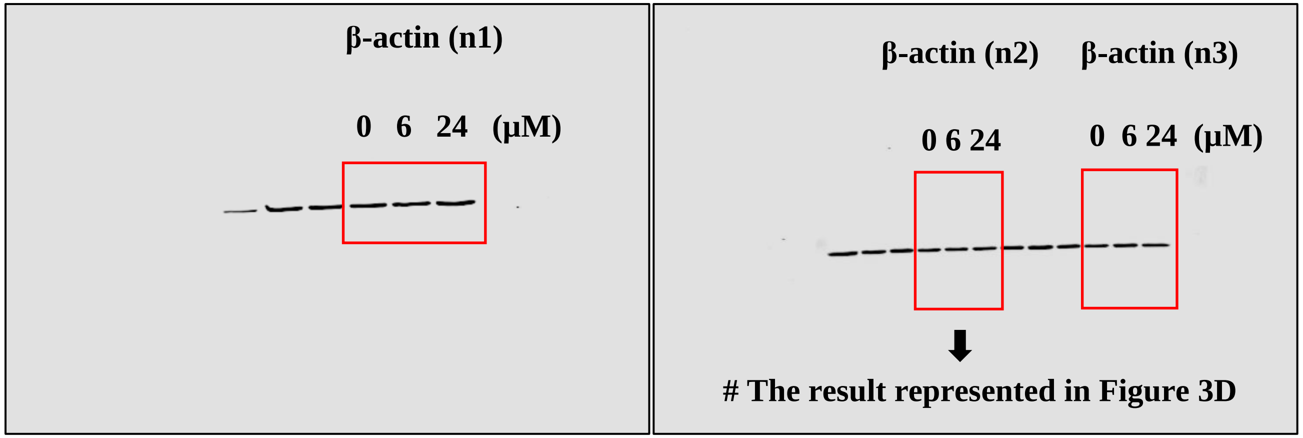

Rb (110 KDa, green) and p-Rb Ser780 (110 KDa, red)

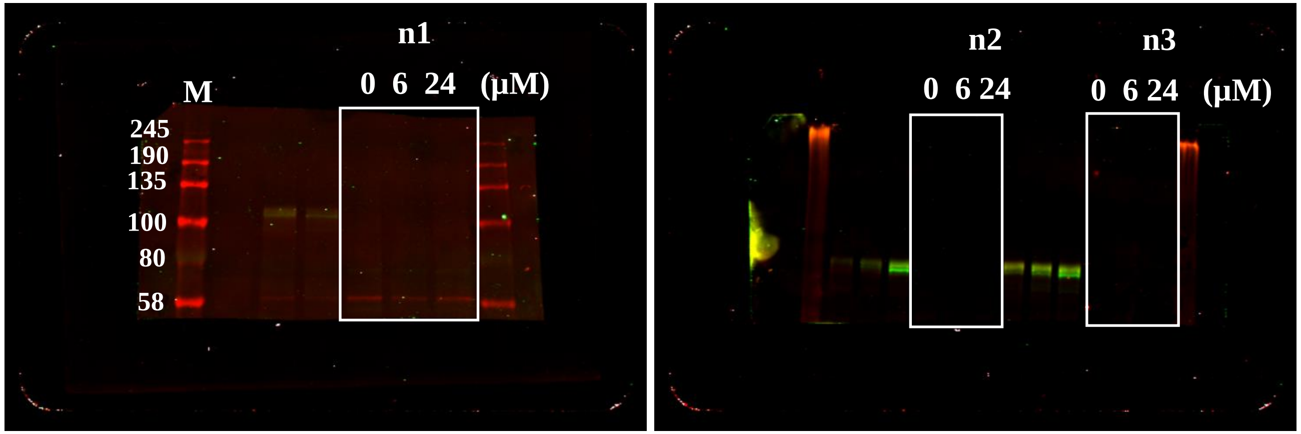

β-actin (42 KDa, green) of Rb and p-Rb

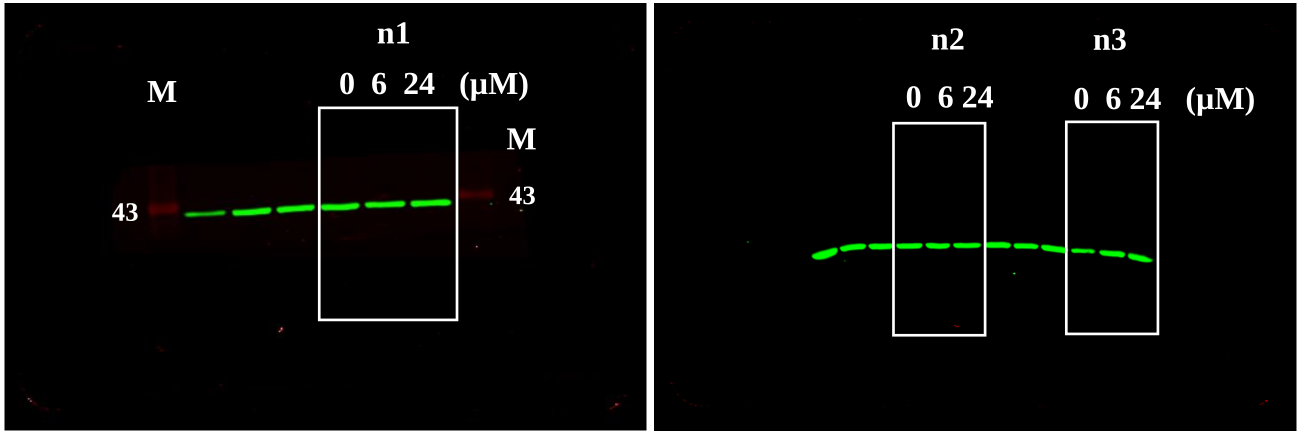

Chk1 (56 KDa, red) and p-Chk1 Ser345 (56 KDa, green)

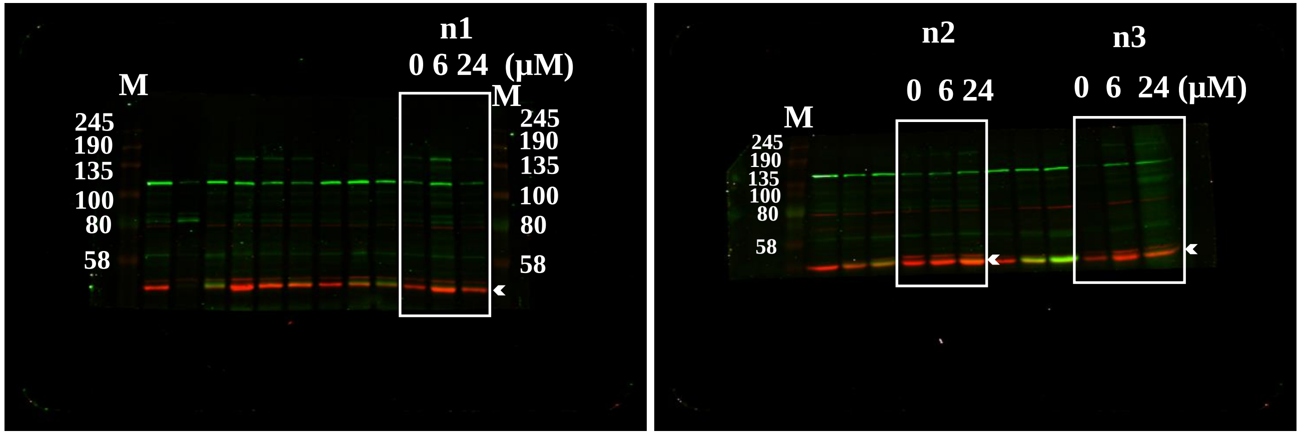

β-actin (42 KDa, green) of Chk1 and p-Chk1

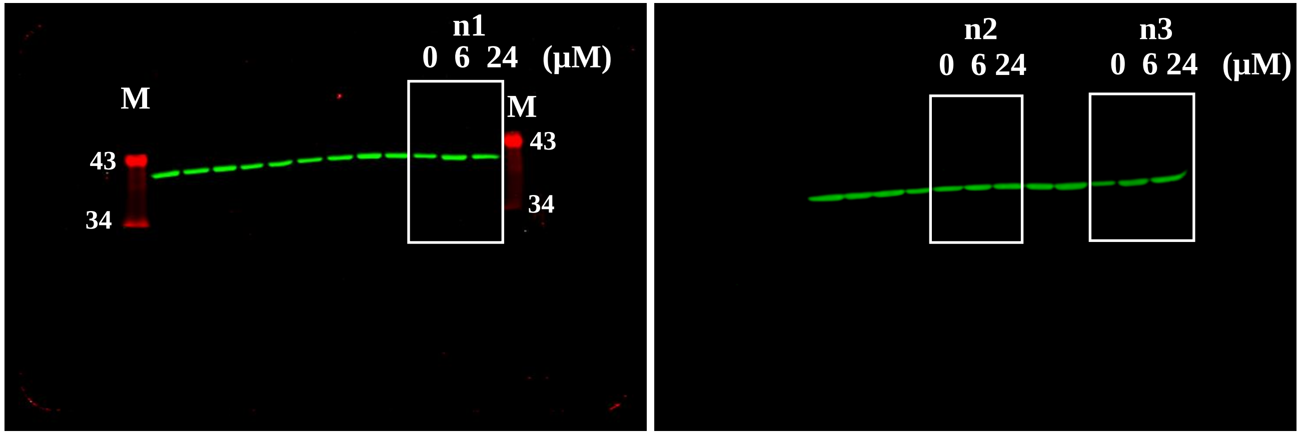

Chk2 (62 KDa, red) and p-Chk2 Ser516 (62 KDa, green)

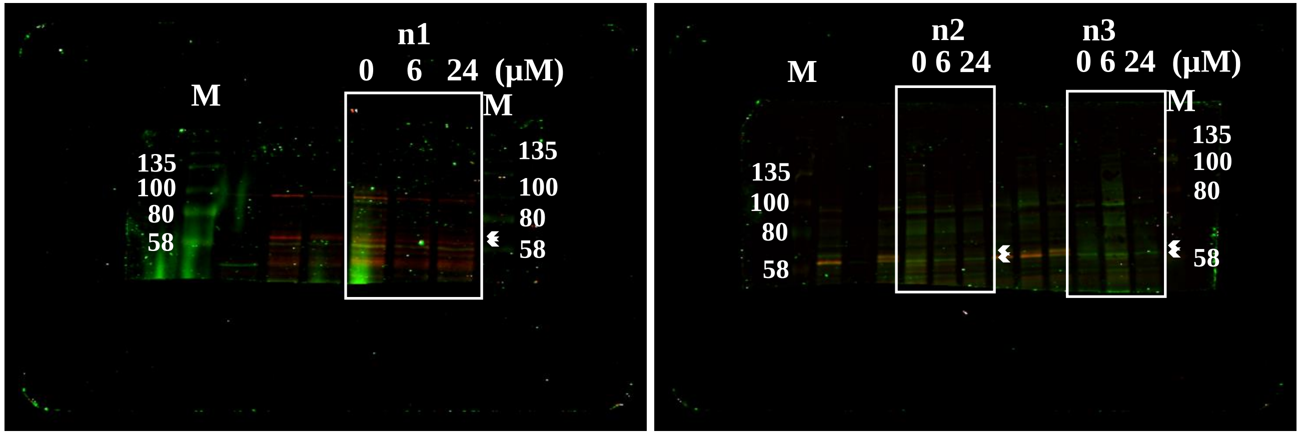

β-actin (42 KDa, green) of Chk2 and p-Chk2

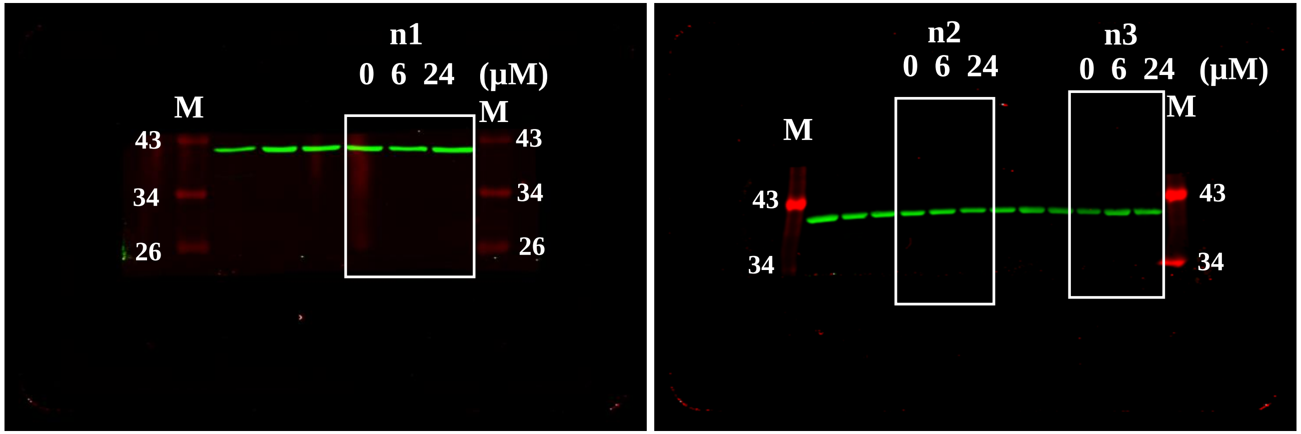

p-H2AX (17 KDa, green)

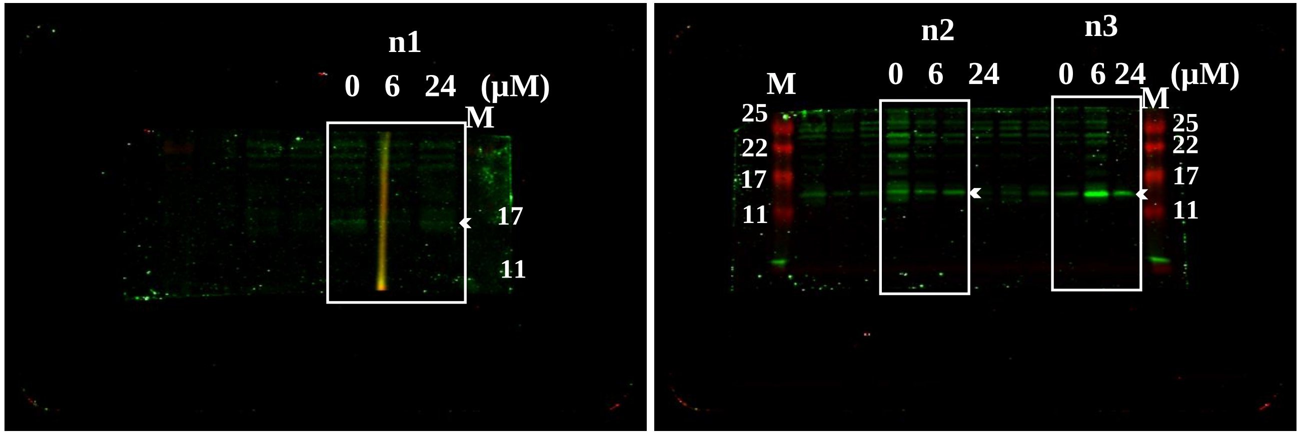

β-actin (42 KDa, green) of p-H2AX

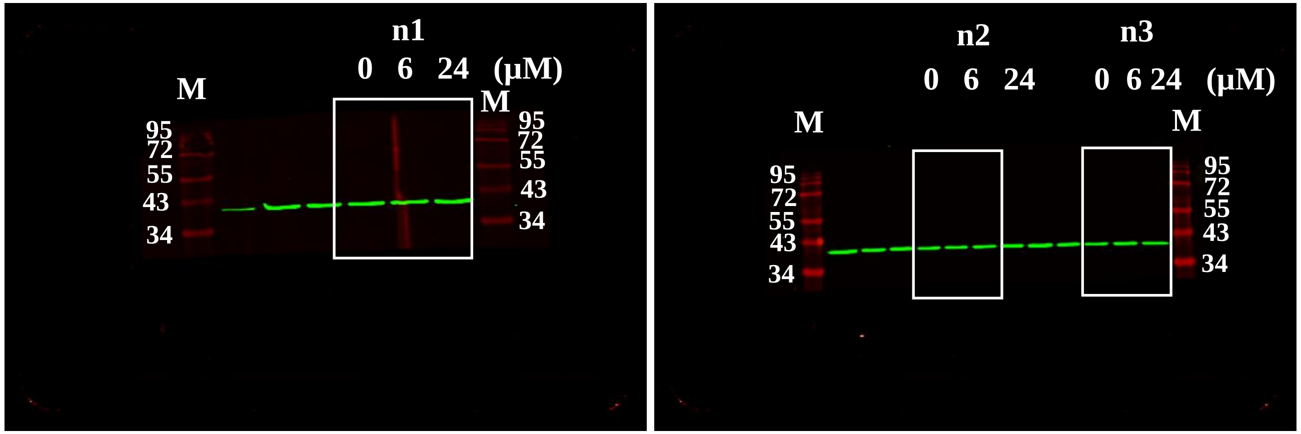

7HF concentration (µM)	Absorbance (268 nm)		
	#1	#2	#3
125.85	0.09184	0.08400	0.05441
251.69	0.34277	0.38235	0.29000
503.38	0.41608	0.44129	0.49000
1,006.77	0.68008	0.64546	0.63684
2,013.53	0.70305	0.73100	0.91060
4,027.06	0.96164	0.97003	0.83350

Table S1: Absorbance of 7HF ranged from 125.85-4,027.06 µM at 268 nm. #1-#3, the sample n1-n3

Time (h)	Absorbance (268 nm)					
	7HF-cell culture			7HF-cell free medium		
	#1	#2	#3	#1	#2	#3
0.00	0.000	0.000	0.000	0.000	0.000	0.000
0.25	-0.037	-0.091	-0.035	0.188	0.256	0.200
0.50	0.008	-0.022	-0.003	0.359	0.333	0.355
0.75	0.205	0.239	0.141	0.249	0.226	0.306
1.00	0.240	0.190	0.211	0.292	0.280	0.312
5.00	-0.024	0.025	-0.004	0.279	0.381	0.363
9.00	-0.181	-0.092	-0.095	0.387	0.332	0.358
13.00	-0.207	-0.209	-0.099	0.196	0.212	0.285
17.00	-0.300	-0.282	-0.177	0.230	0.094	0.172
24.00	-0.269	-0.295	-0.248	-0.104	-0.063	-0.150

Table S2: 1,006.77 µM 7HF was measured at wavelength 268 nm for 0.25-24 h both cell- and cell free condition. #1-#3, the sample n1-n3
